# Inert Pepper aptamer-mediated endogenous mRNA recognition and imaging in living cells

**DOI:** 10.1093/nar/gkac368

**Published:** 2022-05-17

**Authors:** Qi Wang, Feng Xiao, Haomiao Su, Hui Liu, Jinglei Xu, Heng Tang, Shanshan Qin, Zhentian Fang, Ziang Lu, Jian Wu, Xiaocheng Weng, Xiang Zhou

**Affiliations:** College of Chemistry and Molecular Sciences, Key Laboratory of Biomedical Polymers-Ministry of Education, Wuhan University, Luojiashan Street, Wuchang District, Wuhan, HuBei 430072, PR China; College of Chemistry and Molecular Sciences, Key Laboratory of Biomedical Polymers-Ministry of Education, Wuhan University, Luojiashan Street, Wuchang District, Wuhan, HuBei 430072, PR China; College of Chemistry and Molecular Sciences, Key Laboratory of Biomedical Polymers-Ministry of Education, Wuhan University, Luojiashan Street, Wuchang District, Wuhan, HuBei 430072, PR China; Department of Chemistry, Yale University, 225 Prospect Street, New Haven, CT 06520, USA; College of Chemistry and Molecular Sciences, Key Laboratory of Biomedical Polymers-Ministry of Education, Wuhan University, Luojiashan Street, Wuchang District, Wuhan, HuBei 430072, PR China; College of Chemistry and Molecular Sciences, Key Laboratory of Biomedical Polymers-Ministry of Education, Wuhan University, Luojiashan Street, Wuchang District, Wuhan, HuBei 430072, PR China; College of Chemistry and Molecular Sciences, Key Laboratory of Biomedical Polymers-Ministry of Education, Wuhan University, Luojiashan Street, Wuchang District, Wuhan, HuBei 430072, PR China; College of Chemistry and Molecular Sciences, Key Laboratory of Biomedical Polymers-Ministry of Education, Wuhan University, Luojiashan Street, Wuchang District, Wuhan, HuBei 430072, PR China; College of Chemistry and Molecular Sciences, Key Laboratory of Biomedical Polymers-Ministry of Education, Wuhan University, Luojiashan Street, Wuchang District, Wuhan, HuBei 430072, PR China; College of Chemistry and Molecular Sciences, Key Laboratory of Biomedical Polymers-Ministry of Education, Wuhan University, Luojiashan Street, Wuchang District, Wuhan, HuBei 430072, PR China; School of Medicine, Wuhan University, Luojiashan Street, Wuchang District, Wuhan, HuBei 430072, PR China; College of Chemistry and Molecular Sciences, Key Laboratory of Biomedical Polymers-Ministry of Education, Wuhan University, Luojiashan Street, Wuchang District, Wuhan, HuBei 430072, PR China; College of Chemistry and Molecular Sciences, Key Laboratory of Biomedical Polymers-Ministry of Education, Wuhan University, Luojiashan Street, Wuchang District, Wuhan, HuBei 430072, PR China; The Institute of Advanced Studies, Wuhan University, Luojiashan Street, Wuchang District, Wuhan, HuBei 430072, PR China

## Abstract

The development of RNA aptamers/fluorophores system is highly desirable for understanding the dynamic molecular biology of RNAs *in vivo*. Peppers-based imaging systems have been reported and applied for mRNA imaging in living cells. However, the need to insert corresponding RNA aptamer sequences into target RNAs and relatively low fluorescence signal limit its application in endogenous mRNA imaging. Herein, we remolded the original Pepper aptamer and developed a tandem array of inert Pepper (iPepper) fluorescence turn-on system. iPepper allows for efficient and selective imaging of diverse endogenous mRNA species in live cells with minimal agitation of the target mRNAs. We believe iPepper would significantly expand the applications of the aptamer/fluorophore system in endogenous mRNA imaging, and it has the potential to become a powerful tool for real-time studies in living cells and biological processing.

## Graphical Abstract

**Graphical Abstract:**
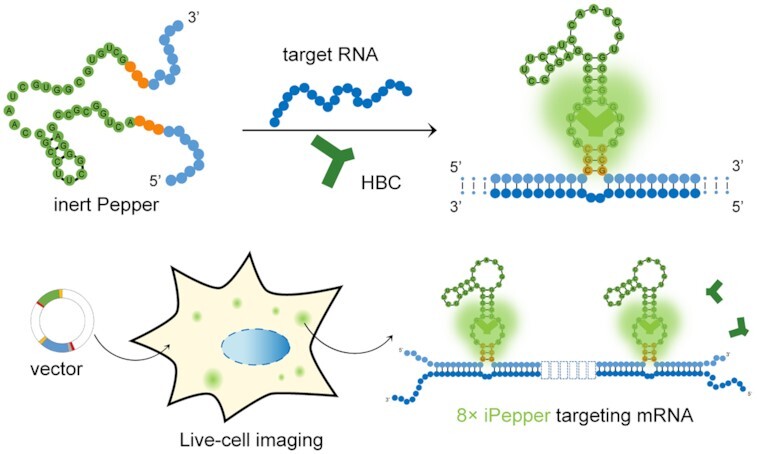
A gene-encoded system is designed for the noninvasive imaging of endogenous RNA in living cells. In the presence of HBC, iPepper with a loose structure does not emit fluorescence until it binds to the target RNA. The fluorescence turn-on mechanism renders this system high efficiency and sensitivity.

## INTRODUCTION

Visualization of the distribution and dynamics of mRNA in living cells provides crucial knowledge about mRNA localization and trafficking, which play essential roles in gene expression and regulation (1–4). An increasing number of methods, such as improved molecular beacons (5–7), quencher-free probes (8–12), and DNA forced intercalation probes, based on fluorescence in situ hybridization (FISH) (13–16) have been developed to image mRNAs in living cells with high sensitivity. Unfortunately, these methods need to synthesize a set of specific probes for each RNA target and usually invade cells site-specifically through microinjection, transfection, or other delivery technologies. Therefore, the progress of universal gene-encoded live-cell mRNA probes is essential to provide the dynamics and distribution of endogenous mRNA.

The RNA binding protein-fluorescent protein system (17–20), represented by the MS2-GFP system, is based on RNA tags and fusion proteins for detecting exogenous mRNA. On this basis, a bimolecular fluorescence complementation system (21–25) was also derived, which splits the fluorescent protein to reduce the fluorescence background. Recently, a series of green fluorescent protein (GFP)-mimicking turn-on RNA aptamers have simplified the mRNA imaging process (26–34). The addition of cell-permeable fluorophore avoids the tedious construction and recruitment of fusion proteins. Nevertheless, these methods require modifying RNA transcripts, such as integrating RNA aptamer sequences into 3′UTRs of the target mRNA, which is a challenge to image non-engineered, endogenous mRNAs. Therefore, researchers have developed several genetically encoded RNA-based fluorescent biosensors (35–47). A representative method is a quencher-fluorophore conjugate light-up aptamer designed by Sato *et al.* (41). The fluorescence of the BHQ-fluorophore conjugate was restored after the target specifically recognizes and binds to the target-binding domain. Fan's group reported an aptamer-initiated fluorescence complementation method of mRNA imaging by dividing the Broccoli aptamer into two split fragments that tandemly bind to target mRNA (38). Both probes are genetically encoded in vectors for cellular expression and allow visualization of non-engineered, endogenous mRNAs in living cells. Nevertheless, direct imaging of low-copy-number mRNAs remains a challenge because of the relatively low brightness of fluorescent RNA aptamers in the cytosolic environment.

**Figure 1.**
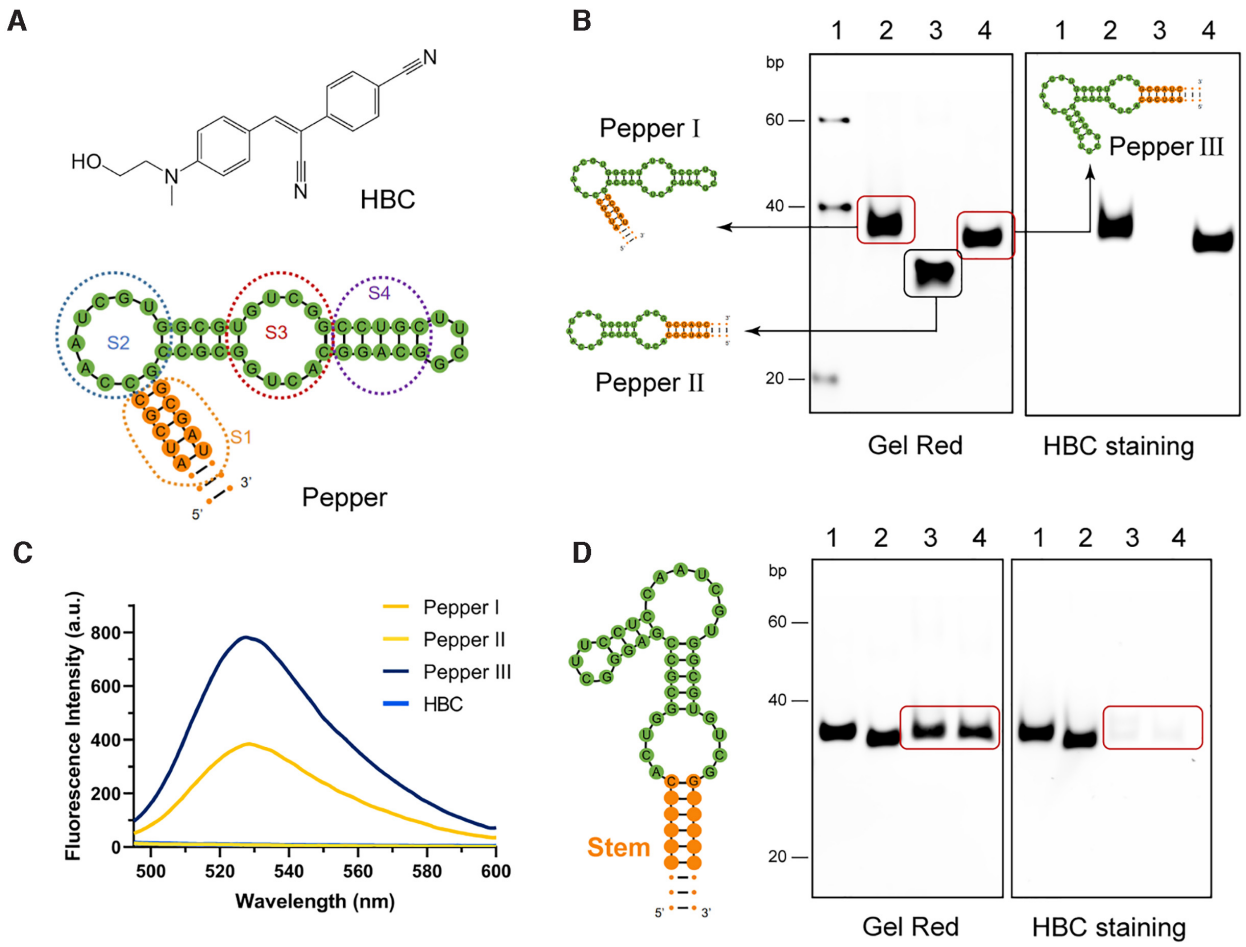
Design and optimization of iPepper aptamers. (**A**) Chemical structure of HBC and predicted secondary structure of Pepper. HBC has no fluorescence in solution but emits strong fluorescence when restricting intramolecular motion (32). Predicted secondary structure of Pepper as previously reported. Green indicates bases in the key parts. Orange indicates the bases that help form the structure. S1 to S4 marked by dashed lines represent the different parts of Pepper. (**B**) 10% PAGE analysis of three Pepper aptamers. Lane 1, 20 bp Marker. Lane 2, Pepper I. Lane 3, Pepper II. Lane 4, Pepper III. Gels were stained first with HBC (right) and subsequently with Gel Red (left). (**C**) Fluorescence spectra of three variants of Pepper aptamer in the presence of 1 μM HBC. Three independent experiments were carried out with similar results. (**D**) 10% PAGE analysis of Pepper III with a stem length of 2–6 bp. Lane 1, Pepper III-6nt; Lane 2, Pepper III-4nt; Lane 3, Pepper III-3nt; Lane 4, Pepper III-2nt. Gels were stained first with HBC (right) and subsequently with Gel Red (left). The corresponding HBC staining band disappears when the stem length of Pepper III is less than 4 bp. The stem is marked in orange. The bands showing differences in the two gel images are circled in red.

The Pepper RNA aptamers exhibit bright and stable fluorescence when combined with the fluorophore-like synthetic dye (32), (4-((2-hydroxyethyl) (methyl)amino)-benzylidene)-cyanophenyl-acetonitrile (HBC, Figure 1A). Among them, Pepper530 exhibits excellent brightness, better than current RNA mimics of GFPs. Simultaneously, Pepper possesses a simpler structure. We assume that by modifying separate parts of the Pepper RNAs’ structure, it will be possible to regulate its fluorescence activity. In this study, we engineered an ‘inert Pepper’ RNA which was destabilized and unfolded due to a very short terminal stem. This modification prevented the binding with its ligand. In the presence of HBC dye, the iPepper with complementary sequences to 5′ and 3′ ends of the targeted RNA can fold correctly by binding to the target RNA. The fluorescence increased upon binding to the target RNA allowing imaging of endogenous RNAs. Consequently, iPepper did not require genetic manipulation of the target RNA. Furthermore, connecting this system in series enables the target recognition system with multiple repeating units to be formed, increasing the sensitivity of mRNA imaging.

## MATERIALS AND METHODS

### General materials and methods

Detailed information concerning the comparison between Split Broccoli system and iPepper system, target selectivity, gene and protein expression levels, real-time imaging of *β-actin* mRNA, NMR data, DNA and RNA sequences are provided in the Supplementary Data (Supplementary Tables S2 and S3).

### Synthesis of HBC

Synthesis of HBC was carried out as previously reported (32). To a stirring solution of *p*-[*N*-methyl-*N*-(beta-hydroxyethyl)]amino benzaldehyde (360 mg, 2 mmol) and 4-cyano-benzeneacetonitrile (620 mg, 4.4 mmol) in 20 ml dry methanol, two drops of piperidine were added. After stirring at ambient temperature for 1 h, the mixture was cool to room temperature, dried under reduced pressure, and column chromatographed (DCM/MeOH 100:1) to give HBC as orange solid (497 mg, 1.64 mmol, 82%). HBC was dissolved to 1 mM with dimethyl sulfoxide (DMSO) and stored at -20°C. ^1^H NMR (400 MHz, DMSO-*d*_6_) δ 7.99 (s, 1H), 7.94–7.81 (m, 6H), 6.88–6.79 (m, 2H), 4.77 (t, *J* = 5.4 Hz, 1H), 3.60 (q, *J* = 5.7 Hz, 2H), 3.53 (t, *J* = 6.2 Hz, 2H), 3.06 (s, 3H). ^13^C NMR (101 MHz, DMSO-*d*_6_) δ 152.0, 145.8, 140.1, 133.3, 132.4, 125.8, 120.5, 119.3, 119.2, 111.9, 110.1, 100.2, 58.7, 54.3, 39.2 (Supplementary Figures S15 and S16).

### Preparation of RNAs for *in vitro* experiments

For 1× aptamer, the ssDNA templates were purchased from Genscript (Nanjing) and annealed to a short T7 primer or complementary sequences (final concentrations 10 μM). The target RNAs were purchased from Genscript (Nanjing). For 2–8× aptamer, the dsDNA templates for aptamer and target were the polymerase chain reaction (PCR) product from the corresponding vectors. For full-length mRNA, the cDNAs of *tubulin beta 3 class III* (*TUBB3)* were amplified from the total RNA of HeLa cells by RT (reverse transcription) with M-MuLV Reverse Transcriptase (NEB) and followed by PCR with FastPfu DNA polymerase (TransGen Biotech). RNAs were prepared by *in vitro* transcription reaction overnight at 37°C using the TranscriptAid T7 High Yield Transcription Kits (Thermo Fisher Scientific). Then, RNAs were purified using RNA Clean & Concentrator™ kits. All RNAs were stored in diethylpyrocarbonate (DEPC)-treated water at 10 μM concentrations in a -80°C refrigerator.

### *In vitro* characterization of iPepper

For the comparison of three variants of Pepper and optimization of the length of the stem region, 1 μM RNA were incubated in imaging buffer (40 mM HEPES, pH 7.5, 100 mM KCl, 5 mM MgCl_2_) containing 1 μM HBC.

For the study of dose-dependent effects of target RNA and HBC, 1 μM iPep III-*β-actin* RNA were incubated with 0, 0.2, 0.5, 1, 2 μM target RNA respectively in imaging buffer containing 2 μM HBC. For dose-dependent effects of HBC, 1 μM iPep III-*β-actin* RNA and 1 μM target RNA were incubated in imaging buffer containing 0, 0.5, 1, 2, 5 and 10 μM HBC, respectively. The group without iPep III-*β-actin* RNA was used as a negative control.

For the selective RNA recognition experiment, 1 μM iPep III-*β-actin* RNA were incubated with 2 μM full-match or mismatch target RNA respectively in imaging buffer containing 2 μM HBC. All samples were annealed by first holding at 65°C for 10 min and then cooling to 25°C at a rate of 0.5°C/30 s. The incubation step was performed with a Bio-Rad T100™ Thermal Cycler, followed by polyacrylamide gel electrophoresis (PAGE) analysis or spectroscopic measurement.

### Comparison between Split Broccoli system and iPepper system

The samples were incubated in imaging buffer and anneal with the above procedure. The fluorescence intensity was recorded with a Bio-Rad CFX96 Touch™ Real-Time PCR Detection System and analyzed with GraphPad Prism. To characterize their respective binding rate with target RNA, samples 6, 7, 15, and 17 in Supplementary Figure S1A were prepared without target RNA. All samples were first denatured by placed at 65°C for 10 min and immediately put on ice for at least one minute. Then, target RNA was added to the corresponding samples and the fluorescence data were collected immediately at 37°C. The final fluorescence intensity was obtained by measuring the fluorescence spectrum after incubation for 18 h.

### *In vitro* characterization of tandem arrays of iPepper

For the combination of tandem arrays of iPepper (1×, 2×, 4× and 8×) with and without target RNA, 200 nM tandem arrays of iPepper were incubated with 400 nM corresponding target RNA in imaging buffer containing 4 μM HBC. For the combination of 8× iPep-*β-actin* aptamer with target RNA, 200 nM 8× iPep-*β-actin* was incubated with 0, 50, 100, 200, 400 and 800 nM target RNA respectively in imaging buffer containing 4 μM HBC. The annealing process was as described above.

### Native polyacrylamide gel electrophoresis (PAGE) analysis

The combination of Pepper and target were analyzed with 10% native PAGE of 1.0 mm thickness in 1× TBE buffer (89 mM Tris, 89 mM boric acid and 2 mM EDTA). The samples were run at 200 V for 2 h in pre-cooled 1× TBE buffer at 4°C. To detect Pepper-HBC complexes, gels were first incubated in imaging buffer (40 mM HEPES, pH 7.5, 100 mM KCl, 5 mM MgCl_2_) containing 10 μM HBC for 15 min and scanned with ChemiDoc MP (Bio-Rad Laboratories) imaging system in Alexa 488 channel. Next, counterstaining with GelRed^®^ was performed to detect all RNA species in each lane. The gels were scanned with ChemiDoc XRS+ (Bio-Rad Laboratories) in a Gel Red channel.

### Spectroscopic measurements

All samples were prepared in the imaging buffer. Fluorescent emission spectra were acquired with PerkinElmer LS 55 (PerkinElmer, USA). Except for special instructions, the excitation wavelength used in all fluorescence tests was 470 nm.

### Cell culture

HeLa, COS-7, HEK293T, U2OS and MCF-7 cells were cultured in Dulbecco's modified Eagle's medium (DMEM; Gibco) supplemented with 100 units/ml penicillin, 100 μg/ml streptomycin sulfate, and 10% fetal bovine serum (Gibco) at 37°C in a humidified 5% CO_2_ incubator. NIH/3T3 cells were cultured in Special medium for NIH/3T3 cells (Procell Life Science & Technology Co., Ltd.) under 37°C with 5% CO_2_.

### Construction of aptamer expression plasmids

All aptamer sequences used to construct expression vectors were derived from gene synthesis (Genscript, Nanjing).

For the imaging of exogenous *β-actin* mRNA, the cDNAs of *β-actin* were amplified from the total RNA of HeLa cells by RT (reverse transcription) with M-MuLV Reverse Transcriptase (NEB) and followed by PCR with FastPfu DNA polymerase (TransGen Biotech), a forward primer (5′-CGCTAGCTAGCACCGCCGAGACCGCGTCC-3′), and a reverse primer (5′-GCTAGTCTAGATGTACAGGTAAGCCCTGG-3′). The cDNAs were incorporated into pcDNA3.1 plasmid with T4 ligase (Thermo Fisher Scientific) after dual digesting with restriction enzymes NheI and XbaI (NEB). The ligated products were transformed into DH5α (TransGen Biotech). The constructed plasmids pcDNA3.1-*β-actin* were selected by DNA sequencing. The dsDNA templates for the corresponding aptamer (1× iPep-*β-actin* and 8× iPep-*β-actin*) were further inserted into the XbaI and EcoRI sites of pcDNA3.1-*β-actin*. For the F30-8× Pepper, the DNA sequence was derived from gene synthesis and cloned into NheI and EcoRI sites of pcDNA3.1.

For the imaging of endogenous *β-actin* and other mRNAs, the BFP sequence was first amplified and incorporated into pLKO5.sgRNA.EFS.tRFP (Addgene #57823) plasmid to replace the RFP gene to produce constructed plasmids pLKO5.sgRNA.EFS.BFP. The dsDNA for the corresponding 8× iPepper aptamers was further cloned into the BmgBI and EcoRI sites (located downstream of the U6 promoter) of pLKO5.sgRNA.EFS.BFP, followed by the process mentioned above.

### Transfection of plasmids encoding RNA aptamer

Cells were seeded on glass-bottom dishes (20 mm) 24 h before transfection. All the cell lines were transiently transfected using Lipofectamine 3000 reagent (Life Technologies) according to the manufacturer's recommendations. The transfected cells were incubated for 30 h before imaging.

### Live-cell imaging

Thirty hours after transfection, cells were incubated with FluoroBrite™ DMEM (Thermo Fisher Scientific) Media or imaging buffer containing 1 μM HBC for 20 min before imaging. The nuclei were stained using 1 μg/ml Hoechst 33342. Images were obtained using Andor Revolution XD confocal laser scanning microscope or Leica TCS SP8 DIVE microscope equipped with an HC PL APO CS2 ×63.0/1.40 OIL objective and gadolinium hybrid (HyD) detectors, with a 488 nm laser excitation. Generally, we choose cells with appropriate and consistent cell density under the brightfield observations. Then cells expressing BFP were located through the DAPI channel to ensure that the vectors were successfully transfected and expressed. The data of all channels of these cells were collected subsequently and comprehensively analyzed by ImageJ software. For each differently treated sample, at least 20 BFP-positive cells were analysed.

### Imaging *β-actin* mRNA after arsenite stress

HeLa cells were seeded on glass-bottom dishes (20 mm) 24 h before transfection. Cells were cotransfected with 500 ng pmCherry-C1-G3BP1 (Miaolingbio) and pLKO5-8ip-*β-actin*-BFP (the coexpression vector encoding both 8× iPep-*β-actin* aptamer RNA and BFP). At 30 h after transfection, cells were incubated in a complete medium supplemented with 500 μM sodium arsenite for 45 min. Cells were then stained and imaged using the above mRNA imaging experiments. In general, after finding a field of view in brightfield with an appropriate and consistent cell density, collect data on cells with BFP signal and significant mCherry signal aggregation following sodium arsenite treatment. For each differently treated sample, at least 20 cells were analysed.

### Imaging *GAPDH* mRNA in cells knocked down for the *GAPDH* mRNA

U2OS cells were seeded on glass-bottom dishes (20 mm) 24 h before transfection. Cells were cotransfected with 80 pmol siRNA (GenePharma, Sense: 5′-UGACCUCAACUACAUGGUUTT-3′, Antisense: 5′-AACCAUGUAGUUGAGGUCATT-3′) and pLKO5-8ip-*GAPDH*-BFP (the coexpression vector encoding both 8× iPep-*GAPDH* aptamer RNA and BFP). At 30 h after transfection, cells were then stained and imaged using the above mRNA imaging experiments.

### Fluorescence in situ hybridization (FISH)

On day 0, HeLa cells were seeded on glass-bottom dishes (20 mm) 24 h before transfection of 1 μg plasmids. On day 2, the cells were fixed with 4% paraformaldehyde in PBS and followed by three times of washing with PBS. The cells were then permeabilized with 0.5% Triton X-100, and then in 70% ethanol at 4°C overnight. On day 3, the cells were washed once and soaked in 2× SSC buffer (300 mM NaCl, 30 mM sodium citrate, pH 7.0) containing 50% formamide at 50°C for 15 min, denature and hydrate the mRNAs on cells. Hybridization was performed with 200 ng of denatured Cy3 or Cy5-labeled DNA probe in hybridization solution (2× SSC, 50% formamide, 10% dextran sulfate, 2 mM vanadyl-ribonucleoside complex, 0.02% RNase-free BSA, 1 mg/ml *E. coli* tRNA) for 24 h at 37°C. On day 4, After washing twice with 50% formamide, 2× SSC at 37°C, the nuclei were stained using 1 μg/ml DAPI. The cells were then washed with 2× SSC for 3 × 5 min and followed by counterstain of the aptamers using 1 μM HBC in 2× SSC at 37°C. The fluorescent signal was imaged using an Andor Revolution XD confocal laser scanning microscope. Typically, the fields with appropriate and consistent cell density are found in brightfield, and data was collected on cells with significant FISH (for exogenous mRNA imaging) or BFP (for endogenous mRNA imaging) signals. For each differently treated sample, at least 20 BFP-positive cells were analysed.

### RT-qPCR

For the effects of 8× iPep-*β-actin* on the expression of *β-actin*, HEK293T cells were seeded in a six-well cell culture plate 24 h before transfection with 2 μg plasmids. Cells were collected at 30 h after transfection. For the test of expression levels of the 8× iPepper, HeLa cells were collected at 6, 12, 24, 36, 48 and 60 h after transfection, respectively. Total RNA of all samples was isolated by adding TRIzol (Invitrogen™) following the manufacturer's instructions. The concentration of the total RNA was quantified with NanoDrop 2000. For RT-qPCR, 20–100 ng total RNA and specific primers (obtained from the PrimerBank) were used for the assay with TransScript Green One-Step RT-qPCR SuperMix (TransGen Biotech) according to the manufacturer's recommendations on a Bio-Rad CFX96 Touch™ Real-Time PCR Detection System. Each sample was detected in three repetitive assays. The data were collected using BioRad CFX manager software.

### Western blotting

HEK293T cells were seeded in a six-well cell culture plate 24 h before transfection with 2 μg plasmids. After 48 h transfection, cells were washed twice with cold PBS and lysed in 100 μl RIPA buffer (50 mM Tris-HCl pH 7.4, 150 mM NaCl, 1.5% NP40, 0.1% SDS, 0.5% sodium deoxycholate, 2 mM MgCl_2_) containing 1× PIC (Halt protease and phosphatase inhibitor cocktail, Pierce) at 4°C for 30 min. Lysates were cleared by centrifugation in 12000 g at 4°C for 30 min. Protein was quantified using a Pierce BCA protein assay kit (Thermo Fisher Scientific) according to the manufacturer's instruction. Equal quantities of protein were mixed with SDS loading buffer, boiled at 95°C for 10 min, and loaded onto SDS PAGE gels. The separated proteins were transferred onto a PVDF membrane (Millipore) in an ice bath at 70 V for 2 h. The PVDF membrane was blocked in TBST (Tris-buffered saline, 0.1% Tween 20) containing 5% (w/v) BSA (Beijing Dingguo changsheng Biotechnology Co., Ltd.) at 37°C for 1 h under agitation. Then the PVDF membrane was incubated with primary antibodies mouse anti-*GAPDH* (Proteintech) or mouse anti-*β-actin* (Proteintech) overnight at 4°C. The blot of protein was washed three times with TBST at room temperature, then incubated with HRP-conjugated Affinipure Goat Anti-Mouse IgG (H + L) (Proteintech) in TBST containing 5% (w/v) BSA at 37°C for 1 h. The blots were washed three times again with TBST at room temperature and imaged on a ChemiDocTM XRS+ Imaging System (Bio-Rad) after incubation with Rhea ECL (US Everbright, Inc).

### Flow cytometric analysis

HeLa cells were transfected with the corresponding plasmids according to the procedure described above. Cells with only Lipofectamine 3000 reagent were used as a control. After 30 h, the cells were digested, concentrated, and washed with PBS solution three times, then resuspended in PBS solution containing 1 μM HBC and 5 mM MgSO_4_. The fluorescence was analyzed using a BD Accuri C6 Flow Cytometry (America). Data processing and analysis were performed through the FlowJo software.

### Cytotoxicity assays

Cells were seeded on 96-well plates at a confluency of 5000 cells per well in 100 μl medium and incubated for 24 h. Then, the medium was replaced with 100 μl of fresh medium added with 0–5 μM HBC or 0–50 μM DFHBI-1T. After incubation for another 24 h, cell survival was evaluated using an MTT assay. 10 μl aliquot of MTT solution (5 mg/ml in PBS) was added to each well. After 4 h of incubation, the medium was replaced and supplemented with 200 μl of DMSO. Absorbance at 492 nm was determined for each well. Data were expressed as mean values of three individual experiments conducted in duplicate.

## RESULTS

### Construction and optimization of iPepper aptamers

The designed aptamer should fold into the correct secondary structure as expected. We used the *NUPACK* web application to forecast the aptamers’ structure and obtain the minimum free energy structure. The fluorescence contribution of each part of the Pepper530 is quite different. When the S3 domain was truncated, the fluorescence intensity decreased the most, which was followed by S2 (Figure 1A). The S1 and S4 domains had almost no contribution to the fluorescence but they help to maintain the integrity of S2 and S3. Hence, domains S1 and S4 were modified to produce three basic structures (Figure 1B). Neutral polyacrylamide gel electrophoresis (PAGE) and fluorescence spectroscopy analysis showed that Pepper I and III maintained their respectable fluorescence activity, while the Pepper II did not (Figure 1B). Pepper III exhibited about twice higher fluorescence intensity than Pepper I under *in vitro* conditions (Figure 1C).

Based on the good fluorescence intensity of Pepper III, we designed Peppers with a stem of 2–6 bp. The HBC staining band disappeared when its stem length was < 4 bp (Figure 1D). Under the test conditions, the intramolecular hydrogen bonds of the 3 bp-stem were thermodynamically unstable. Pepper III tended to form a loop structure without fluorescence activity in the case when the fluorescence activity was not detected. In other words, the 3 bp-stem was considered the equilibrium point for the Pepper III to form a functional structure, at which it would fold into a functional conformation and restore its fluorescence in the presence of the applied external factors (target RNA).

### iPeppers achieved activation of the fluorescence effect *in vitro*

**Figure 2.**
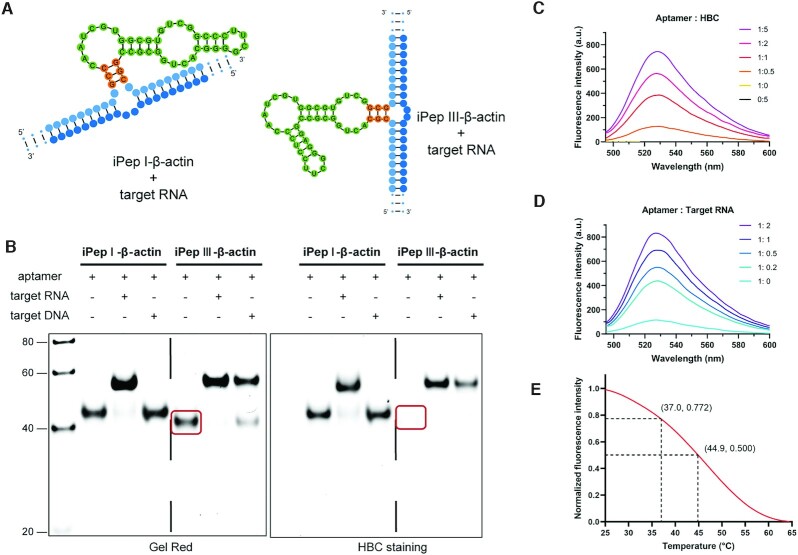
Characterization and condition optimization of iPepper aptamers. (**A**) Predicted secondary structure of iPepper combined with target RNA. iPep I-*β-actin* and iPep III-*β-actin* were designed based on Pepper I and III respectively. (**B**) 10% PAGE analysis of iPepper aptamer binding with target RNA. Gels were stained first with HBC (right) and subsequently with Gel Red (left). The lane of iPep III-*β-actin* without target RNA stained by HBC showed no corresponding band circled in red, while the iPep I-*β-actin* shows the opposite result. (**C**) Fluorescence spectra of iPepper (iPep III-*β-actin*) with different HBC equivalents. The sample with a ratio of iPepper to HBC of 0:5 served as a negative control. (**D**) Fluorescence spectra of iPepper (iPep III-*β-actin*) with different target RNA equivalents. (**E**) Temperature-dependence normalized fluorescence intensity of iPep III-*β-actin* aptamer and target RNA with 1 μM HBC. The fluorescence decreased to 22.8% and 50% of the initial value at 37.0°C and 44.9°C, respectively.

To verify whether the reconstructed Pepper restores its fluorescence activity after targeting, we designed two iPepper aptamers to target *β-actin* mRNA, the iPep I-*β-actin* and iPep III-*β-actin*, based on Pepper I and Pepper III, respectively. Specifically, the 5′-end and 3′-end of the two Peppers had sequences complementary to *β-actin* mRNA (Figure 2A). We observed a migration delay rate band, which appeared in the presence of iPep III-*β-actin* and targeted RNA (Figure 2B), indicating the combination of iPep-*β-actin* with the target RNA. Meanwhile, iPep III-*β-actin* did not demonstrate any fluorescence intensity after HBC staining. As a result, iPeppers achieved activation of the fluorescence effect *in vitro*. Alternatively, iPep I-*β-actin* still exhibited a considerable level of background fluorescence intensity. Interestingly, compared to the RNA target, the reduced or almost none binding of the DNA target to aptamers was probably due to the weaker binding force between RNA and DNA (Figure 2B). The target RNA and HBC dye enhanced the fluorescence in a dose-dependent manner (Figure 2C, D). The iPep-*β-actin* aptamer induced at least a 10-fold fluorescence enhancement in the presence of the target RNA, whereas HBC was nearly unresponsive to iPep-*β-actin* alone (Figure 2D). Additionally, the fluorescence intensity of iPep-*β-actin* at 37°C decreased by 22.8% of the initial fluorescence value at 25°C (Figure 2E). This fact led rationally to the design of Pepper and the adjustment of the stem length converting it into an inert aptamer that was bonded specifically to the target mRNA. Thus, it restored its functional structure and activated fluorescence. Moreover, iPepper demonstrated a higher binding rate with the targeted RNA and stronger fluorescence brightness compared to the Split Broccoli system (Supplementary Figure S1). Both HBC and DFHBI-1T showed negligible cytotoxicity to the cells that underwent subsequent experimental conditions (Supplementary Figure S2). The obtained results implied that iPepper produced faster fluorescent signals and generated a stronger fluorescent signal at a lower concentration of fluorophore, which was essential for mRNA sensors influencing its role in a complex cytoplasmic environment.

Sequence selectivity is essential for imaging a specific mRNA in living cells. Therefore, we tested whether the iPep-*β-actin* aptamer discriminates target RNA with partial nucleotide mutations. A perfectly matched sequence produced the strongest fluorescent signal, while none of the other target sequences with incomplete matches or several base mismatches restored the fluorescence of the aptamer (Supplementary Figure S3). These results indicate that the correct folding of iPepper structure, especially the formation of the stem structure, largely depends on the sequence of the target RNA.

### Construction of iPepper tandem array and imaging of exogenous *β-actin*

**Figure 3.**
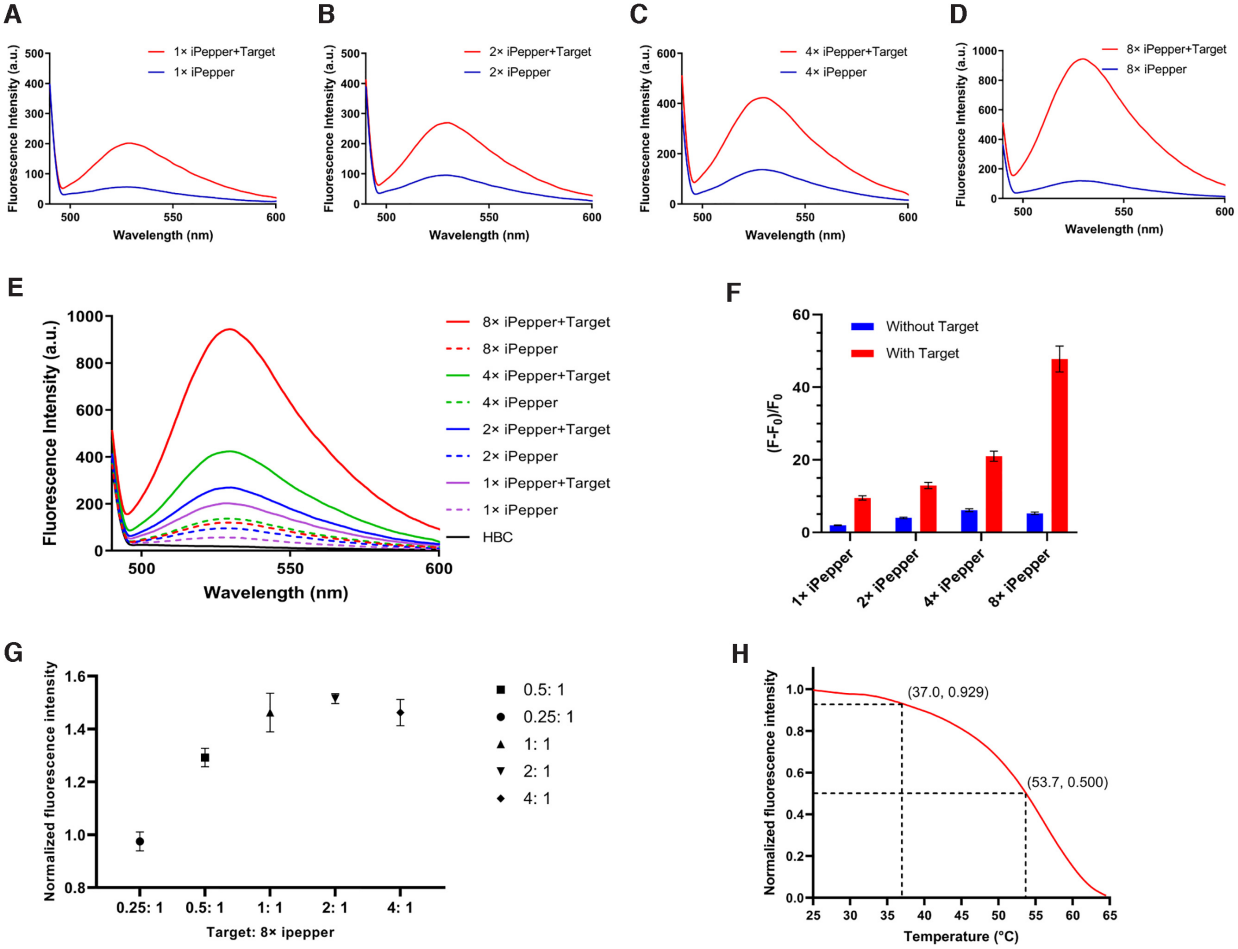
Characterization of iPepper tandem arrays. Fluorescence spectroscopy of 1× (**A**), 2× (**B**), 4× (**C**) and 8× iPepper (**D**) with and without target RNA (*β-actin*). (**E**) The collection of the above fluorescence spectrum. (**F**) Relative fluorescent intensity of different tandem arrays of iPepper (iPep-*β-actin*) aptamer with and without target RNA. Error bars are standard deviations in three repetitive assays. (**G**) Combination of 8× iPep-*β-actin* aptamer with target RNA. 8× iPep-*β-actin* aptamers were incubated with different ratios of target RNA. Fluorescence was measured after annealing. When target: 8× iPepper = 1:1, the fluorescence signal reaches the platform. The process was equipped with the FAM scanning channel. Error bars are standard deviations in three repetitive assays. (**H**) Temperature-dependence normalized fluorescence intensity of 8× iPep-*β-actin* aptamer and target RNA with 4 μM HBC. The fluorescence decreased to 7.1% and 50% of the initial value at 37.0°C and 53.7°C, respectively.

Following the above ideas, we further constructed a series of tandem arrays containing 2, 4 and 8 iPepper units (2×, 4× and 8× iPeppers), targeting partial sequences of *β-actin* mRNA with complementary lengths of 40, 80 and 160 nt, respectively. The selected target sequence region has been known for its high-efficiency siRNA site located in the last exon region of *β-actin* mRNA (48). Interestingly, when the number of cascades increased up to eight, the fluorescence intensity and signal-to-noise ratio of aptamers increased also under the same conditions (Figure 3A–F). To demonstrate the efficiency of trans-hybridization of the 8× iPep RNA relative to the short RNA targets, the full-length *TUBB3* mRNA target (Supplementary Figure S4A), 1–8× target (partial sequences of *TUBB3* mRNA) and its corresponding 1–8× iPeppers were transcribed *in vitro*. First, both short and full-length mRNA targets bound successfully to all the iPeppers. Meanwhile, the fluorescence of iPeppers attached to short targets was slightly stronger than that of full-length targets. Second, compared to the fluorescence intensity of 2× and 4× iPeppers, this of the 8× iPepper attached to the full-length target demonstrated a decreased fluorescence concerning that of the short target (Supplementary Figure S4B). Most importantly, 8× iPepper exhibited the highest fluorescence intensity and signal-to-noise ratio among all iPeppers. Therefore, we assume that the 8× iPeppers would be effective in detecting full-length targets. The main reason may be that a greater number of cascades were more favorable for ligand-driven folding after binding to the full-length target. When the ratio of the aptamer to target reached 1:1, the fluorescence intensity showed up a plateau (Figure 3G). Additionally, the fluorescence intensity of 8× iPepper at 37°C decreased by only 7.1% of the initial fluorescence value at 25°C *in vitro* (Figure 3H). The reported results indicated that the construction of iPepper tandem array comprehensively improved the effect of a single iPepper molecule fluorescence imaging.

**Figure 4.**
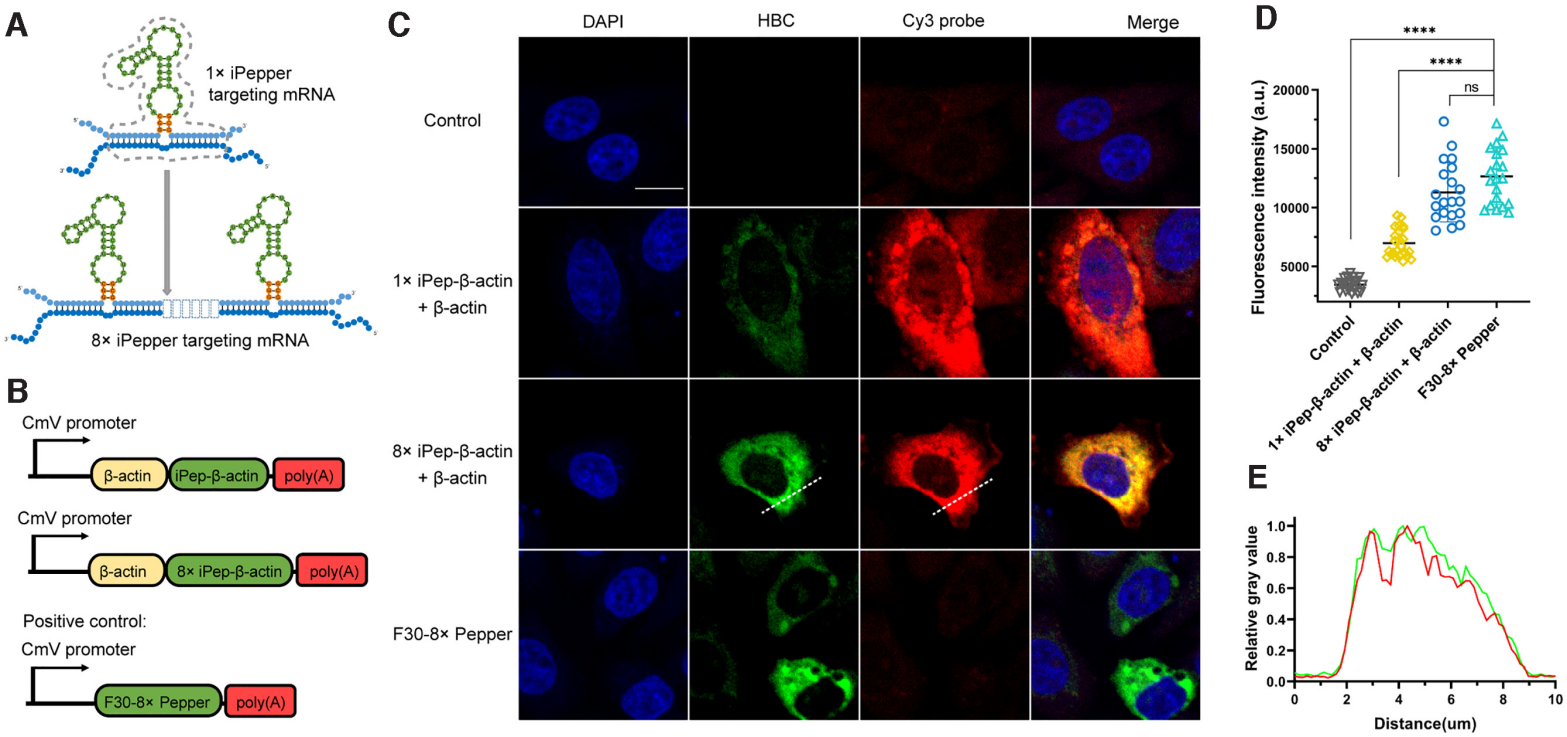
Imaging exogenous *β-actin* mRNA with a genetically encoded iPepper aptamer. (**A**) Schematic representation of eight tandem arrays of iPepper, 8× iPep-*β-actin*. Dark blue represents the targeted mRNA. (**B**) Scheme of plasmids that encode 1× or 8× iPep-*β-actin* in the 3′-end of *β-actin*, and the positive control, F30-8× Pepper, respectively. (**C**) Confocal imaging of fixed HeLa cells coexpressing β-actin and iPepper (1× iPep-*β-actin* and 8× iPep-*β-actin*) in the presence of 1 μM HBC. F30-8× Pepper was used as a positive control. *β-actin* mRNA was co-labeled with Cy3-DNA probe (red) using FISH in fixed HeLa cells. The nucleus is shown in blue (DAPI). Scale bar = 10 μm. Three independent experiments were carried out. (**D**) Mean flourscence indensity of control, 1× iPep-*β-actin* with *β-actin*, 8× iPep-*β-actin* with *β-actin* and F30-8× Pepper in individual cells. Data represent the mean ± s.d. (N = 20 cells). A two-tailed t-test was performed with α = 0.05. NS means not significant, P-value < 0.0001 (****) means a statistically significant. (**E**) The change in relative fluorescence intensity on the straight line marked in (C). The brightest fluorescence intensity on the straight line is counted as 1.0.

The aforementioned tandem arrays showcased multiple iPepper units, which combined with the corresponding target formed Pepper structures, which resulted in the activation of fluorescence (Figure 4A). To determine whether iPepper can image target mRNA in cells, we exogenously transfected a vector encoding *β-actin* mRNA fused with iPep-*β-actin* or 8× iPep-*β-actin* at the 3′-end (Figure 4B). F30-8× Pepper, an 8× Pepper with an F30 scaffold (32), was used as a positive control. 8× iPep-*β-actin* exhibited robust green fluorescence similar to the F30-8× Pepper, and much higher than iPep-*β-actin*, whereas negligible fluorescent signals were observed in control cells (Figure 4C, D). To further verify the binding of inert aptamers and *β-actin* mRNAs in HeLa cells, FISH experiments were carried out. Perfect colocalizations of the green (8× iPep-*β-actin*) and red (Cy3-labeled probe) signals were observed in 90% of successfully transfected cells at least (Figure 4C, E). We also scored the percentage of Cy3-labeled probe signals colocalizing with 8× iPep-*β-actin*. The Manders’ colocalization coefficient data (1× iPep-*β-actin*: *M*_1_= 0.963, *M*_2_= 0.987, 8× iPep-*β-actin*: *M*_1_ = 0.996, *M*_2_ = 0.917) showed a remarkable colocalization relationship between the two probes (Supplementary Figure S5), confirming the observed signals as *β-actin* mRNA.

### Imaging of endogenous *β-actin* mRNA using iPepper

**Figure 5.**
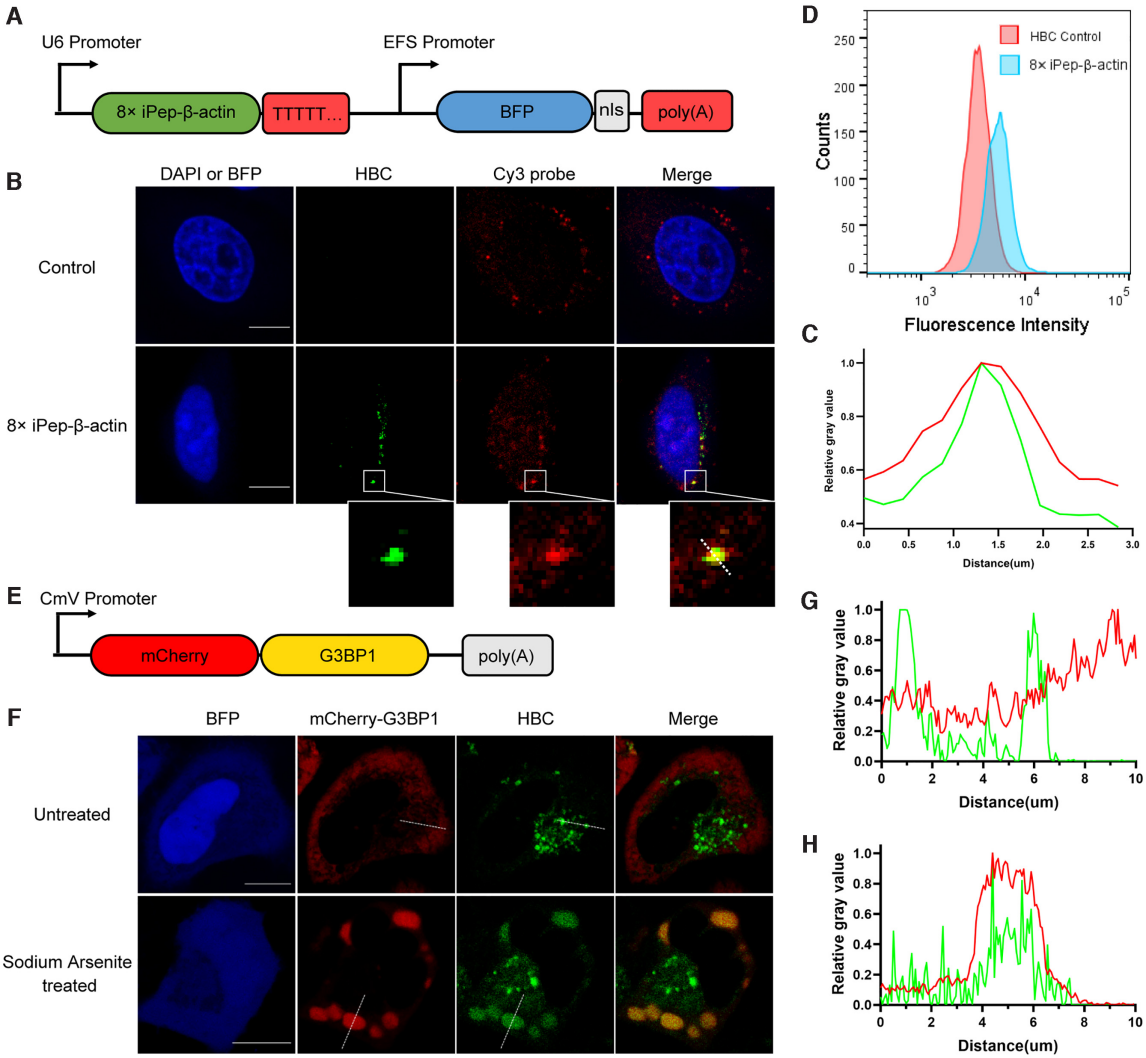
Imaging endogenous *β-actin* mRNA. (**A**) Design and construction of a coexpression vector encoding both 8× iPep-*β-actin* aptamer RNA (green) and BFP (blue). The terminator are shown in red. The part in gray represents the nuclear localization signal. BFP is used as a marker for the expression. (**B**) Fixed HeLa cells with and without transfection were treated with the Cy3 probe targeting *β-actin*, and then imaged in the presence of 1 μM HBC. (**C**) The change in relative fluorescence intensity on the straight line marked in the partial picture in (B). The brightest fluorescence intensity on the straight line is counted as 1.0. (**D**) Flow cytometry histogram of HeLa cells transfected with coexpression vector in (A) (blue) and HBC control cells (red). (**E**) Scheme of plasmids that encode mCherry fused to G3BP1. (**F**) HeLa cells cotransfected with 8× iPep-*β-actin* aptamers and mCherry-G3BP1 fusion proteins in the absence or presence of treatment by sodium arsenite, and then imaged in the presence of 1 μM HBC. (**G, H**) The change in relative fluorescence intensity on the straight line marked in the partial picture in (F), Untreated (G) and Sodium Arsenite treated (H). The brightest fluorescence intensity on the straight line is counted as 1.0. The confocal images show DAPI, BFP (blue), aptamers (green), and Cy3 probes (red). Scale bar = 10 μm. For (B), (D) and (F), at least two independent experiments were carried out with similar results.

Encouraged by the above results, we applied iPepper tandem array to endogenous mRNA in live-cell imaging. To this end, an RNA and protein coexpression vector encoding 8× iPep-*β-actin* and blue fluorescent protein (BFP) was constructed (Figure 5A). BFP RNA can be expressed separately from the aptamer to avoid adverse effects on the folding of the aptamer.

Real-time quantitative reverse transcription PCR (RT-qPCR) was performed to validate the expression of 8× iPep-*β-actin* in cells. The level of 8× iPep-*β-actin* had reached a peak at 12 h after its transfection and remained relatively stable within 60 h (Supplementary Figure S6). RNA and protein levels of *β-actin* were also analyzed. The obtained data did not show any detectable influences on the level of *β-actin* mRNA and protein (Supplementary Figure S7). After transfection, we observed that about 50% of cells had great BFP signals in the nucleus. We confirmed the identity of the *β-actin* mRNA granules using FISH experiments in fixed HeLa cells. Among BFP-positive cells, about 90% exhibited bright HBC signals. Most of the fluorescence signals overlapped with those of a Cy3-labeled probe targeting *β-actin*, which hybridized at a different site from the site targeted by 8× iPep-*β-actin* (Figure 5B, C). Additionally, the flow cytometry also confirmed the increase of fluorescence in living cells (Figure 5D).

The same results were observed when we applied iPepper tandem array to different kinds of living cells. Bright fluorescent spots were observed in the cytoplasm of HeLa, U2OS, HEK293T, MCF-7 and COS-7 cells (Supplementary Figure S8). We observed similar phenomena in 80% of BFP signal-positive cells. The mean fluorescence intensities (MFI) were recorded by flow cytometry and compared to the known expression levels of *β-actin* in four human cell lines. The relative expression of *β-actin* mRNA was derived from *Human Protein Atlas database*. The iPepper imaging results could reflect the mRNA expression to a certain extent (Supplementary Figure S9). However, this did not apply to all cell types, which might be due to differences in the fluorescent background exhibited by different cells in HBC and differences in the transfection and expression efficiency of expression vectors in diverse cell lines. In addition, we performed real-time imaging of *β-actin* mRNA in HeLa cells. Time-lapse images revealed the phenomenon of mRNA particles moving in restricted areas of the cytoplasm (Supplementary Figure S10 and Video S).

Furthermore, 8× iPep-*β-actin* aptamer was co-expressed with mCherry-G3BP1 fusion protein in HeLa cells (Figure 5E), following treatment with sodium arsenite, which induced the formation of stress granules (SGs) (40,49). After transfection, almost all cells with mCherry positive expression in the field of view exhibited diffuse mCherry-G3BP1 signals in the whole area of the cytoplasm and iPepper signals in certain areas of the cytoplasm (Figure 5F, G). After the application of 500 μM sodium arsenite, the dispersed mCherry-G3BP1 fluorescent signal rapidly accumulated, which symbolized the formation of SGs. Meanwhile, partial green fluorescent puncta aggregated to the SGs and perfectly colocalized with mCherry-G3BP1 (Figure 5F, H). That fact further confirmed the identity of the green fluorescent foci, which was *β-actin*.

### Intracellular RNA imaging with iPepper Tandem System exhibits good specificity

**Figure 6.**
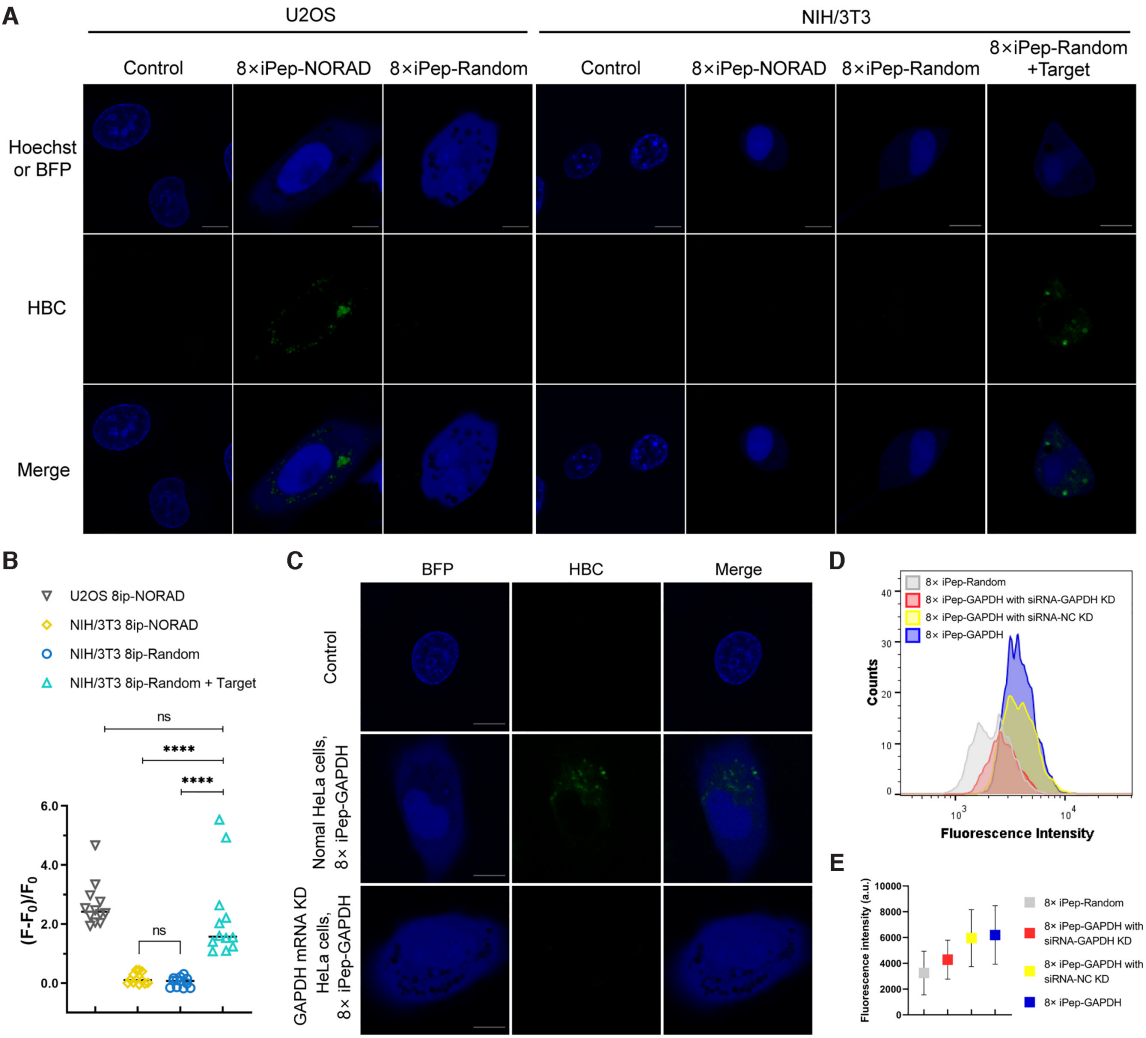
Fluorescence images for U2OS cells and NIH/3T3 cells expressing 8× iPep-*NORAD* or 8× iPep-Random respectively, and NIH/3T3 cells expressing 8× iPep-Random with target RNA transfection. Scale bar = 10 μm. (**B**) Semi-quantitative analysis of 8× iPep-*NORAD* fluorescence in individual U2OS and NIH/3T3 cells and 8× iPep-Random fluorescence with or without target RNA in individual NIH/3T3 cells. Data represent the mean ± s.d. (*N* = 12 cells). A two-tailed t-test was performed with α = 0.05. NS means not significant, *P*-value < 0.0001 (****) means a statistically significant. (**C**) Imaging endogenous *GAPDH* mRNA using 8× iPep-*GAPDH* in normal U2OS cells or U2OS cells with *GAPDH* mRNA knocking down by siRNA. Scale bar = 10 μm. (**D**) Flow cytometry histogram of U2OS cells expressing 8× iPep-*GAPDH* under the treatment of siRNA targeting *GAPDH* (red) and siRNA-NC (yellow). Normal HeLa cells expressing 8× iPep-Random (gray) or 8× iPep-*GAPDH* (purple) without treatment of siRNA were used as the negative control and positive control, respectively. (**E**) The mean fluorescence intensities (MFI) of cell populations in flow cytometry experiment in (D).For (A) and (C), at least two independent experiments were carried out with similar results.

Fluorescence leakage of free mRNA trans-hybridization probes and nonspecifically attached probes in the cytoplasmic environment led to a high background fluorescence and unexpected false-positive results (38). Additionally, we assume that the intracellular environment led to the folding of the ligand-driven aptamer, which may compete with the folding of the target-driven aptamer. To rule out this possibility, an 8× iPep-Random targeting the scrambled control sequence, which is not expected to hybridize to any cellular RNA, was also constructed as a negative control. After validating its target-binding-mediated fluorescence-on function *in vitro* (Supplementary Figure S11), the 8× iPep-Random was expressed in cells as random sequence control. Expectedly, we did not observe any obvious fluorescence signal in both U2OS and NIH/3T3 cells (Figure 6A, B). About 70–90% of BFP-positive cells showed similar results. Further, after exogenous transfection of the random target, at least 50% of NIH/3T3 cells with BFP expression displayed corresponding fluorescent signals in the cytoplasm.

We then attempted to verify if the iPepper tandem array system exhibited fluorescence only when the corresponding target was present in the cell. The abundantly expressed in human cells NORAD lncRNAs were selected, from which the RNA sequence was significantly different from the NORAD in murine cells (Supplementary Figure S12). We designed an 8× iPep-NORAD to target human NORAD RNA. 8× iPep-NORAD illustrated corresponding fluorescent spots in the cytoplasm of U2OS cells, whereas in NIH/3T3 cells there were no obvious fluorescent spots (Figure 6A, B).

Finally, we further evaluated whether the iPepper tandem array system enabled specific imaging of *GAPDH* mRNA in mammalian cells. In U2OS cells with knocked-down *GAPDH* mRNA using siRNA, we noticed few bright iPepper fluorescent spots compared to that in normal U2OS cells (Figure 6C). At least 90% of BFP-positive cells showed similar results. At the same time, the results from the flow cytometry technique showed that the mean fluorescence intensity of U2OS cells in the experimental group decreased to the same level as that in the untreated group and the negative control group treated with siRNA negative to *GAPDH* mRNA (Figure 6D, E). Moreover, RT-qPCR confirmed high knocking-down levels (Supplementary Figure S13). Taken together, these data indicated that the specific binding of iPepper to the target RNA produced the fluorescence signal and that the detected intensity was positively correlated with the content of endogenous target RNA in cells.

### Imaging of endogenous mRNA with low expression levels in living cells

**Figure 7.**
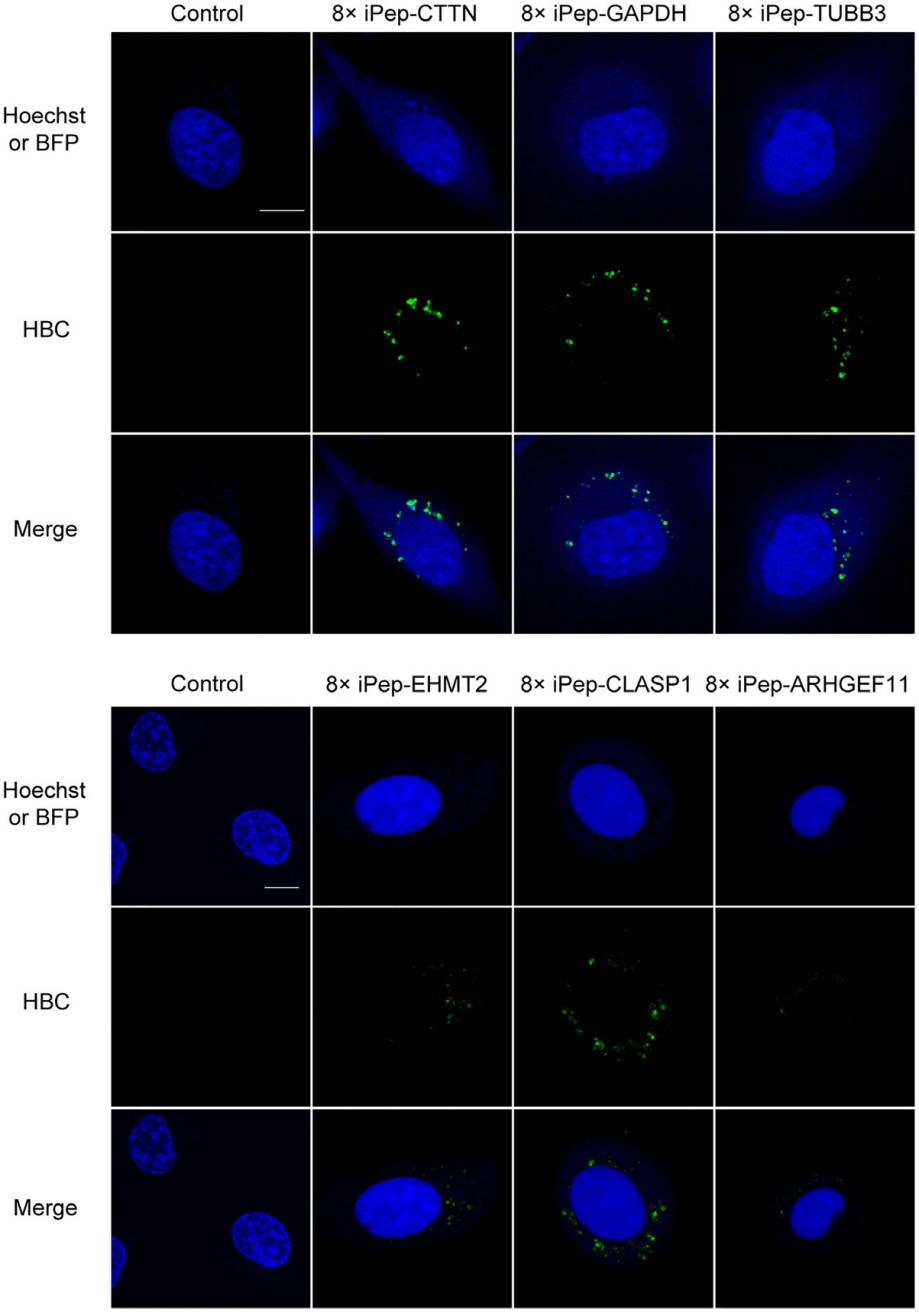
Visualization of *CTTN*, *GAPDH*, *TUBB3*, *EHMT2*, *CLASP1* and *ARHGEF11* mRNA in a single live U2OS cell expressing a coexpression vector encoding iPepper targeting the corresponding mRNA. The fluorescent images show Hoechst 33342, BFP (blue), and aptamers (green). Scale bar = 10 μm. At least three independent experiments were carried out with similar results.

Imaging mRNA with low expression levels has always been a great challenge. On the one hand, the previous fluorescent RNA aptamer (compared to GFP) in the cytosolic environment had relatively low brightness. On the other hand, the background signal of unbound target probes seriously hindered mRNA imaging. In this study, we designed iPepper aptamers targeting six endogenous mRNAs, including *glyceraldehyde-3-phosphate dehydrogenase* (*GAPDH*), *cortactin* (*CTTN*), relatively low expression levels of *euchromatic histone lysine methyltransferase 2* (*EHMT2*), *cytoplasmic linker associated protein 1* (*CLASP1*), *tubulin beta 3 class III* (*TUBB3*), and *Rho guanine nucleotide exchange factor 11* (*ARHGEF11*) (Supplementary Figure S14). All iPeppers’ targets were chosen in the 3′UTR region, with a length of 160 nt. Bright fluorescent spots representing different targeted mRNAs were detected in living U2OS cells (Figure 7). Approximately 90% of BFP-positive cells exhibited similar results. Surprisingly, the iPepper tandem system showed that the *ARHGEF11*, the lowest-expressed mRNA in this study (relative to 0.1% of *GAPDH*), showcased found bright spots in living cells, although it was darker than other highly expressed mRNAs. These results indicated the applicability of iPepper for imaging different endogenous mRNAs in various cells. When selecting target sequences for RNA imaging, it is important to avoid targeting sequences that are ‘buried’ within the tertiary structure or where double-stranded RNA is formed (50). We can predict the sequence of exposed RNA sites by software (e.g. *RNAfold*), but the actual situation in the cell is more complicated, which undoubtedly increases the difficulty of targeted sequences selection. Unexpectedly, all 8× iPepper aptamers targeting mRNAs showed good cellular imaging results when we designed them for the first time. This may be due to the iPepper array system having more aptamer units and longer targeting sequences.

## DISCUSSION

In conclusion, we developed a simple but robust live-cell mRNA imaging system based on Pepper RNA aptamers, called inert Pepper RNAs. We proved that the designed by us system remained inactivated due to its incomplete structure until it hybridized to a specific region of mRNA to restore its complete conformation, resulting in the activation of fluorescence. This universal encoded RNA fluorescence turn-on system noninvasively enabled the imaging of endogenous mRNA without modifying RNA transcripts. Furthermore, we connected this system in series to develop a target recognition system with eight repeating units, realizing highly sensitive mRNA imaging. We strongly believe that these novel sensors are widely applicable for live-cell imaging and provide new ideas for observing relatively low expression levels of the target RNA. To the best of our knowledge, this is the first time it has been demonstrated that certain Pepper RNAs have dual functions of targeting and potentiating fluorescence. Moreover, it is also the first time that multiple inert Pepper RNAs have been connected in series to form a structure complementary to the target for endogenous mRNA imaging in living cells. These findings provide a very possible scenario for the future of the developed by us system with the original RNA aptamer/fluorophore system vast application as a robust tool for RNA imaging.

While imaging methods for fluorescently labeled probes (such as molecular beacons, FRET, quenchers, pure labels, etc.) seem more straightforward, defects such as fluorescence leakage and nonspecific binding cannot be ignored (12). The light-up aptamer approach provides a new idea for solving fluorescence leakage and nonspecific binding. On this basis, our method further correlates the target recognition function of the aptamer with the correct folding ability of the aptamer, that is, wrong target recognition and folding, resulting in the inability of HBC to bind to the aptamer.

The new method offers the following advantages: (i) there is no need to modify the target mRNA or transfection of a fluorescence oligonucleotide chain; (ii) this system combines all the advantages of HBC, such as low-molecular-weight, good membrane permeability, low cytotoxicity, and limited fluorescence in solution or living cells; (iii) the iPepper aptamer array is continuous and complete, which allows avoiding the waste of aptamer resources and greatly enhance the fluorescence signal. On the one hand, multiple aptamer units and longer target binding regions possibly improve the fault tolerance rate of target sequence selection and increase the target accessibility of the system. On the other hand, the system requires sophisticated sequence design and rational structure prediction to ensure low background signals. Although no significant changes in transcription, translation level and cell morphology have been observed in the imaging experiment of *β-actin*, whether such a length of aptamer will affect the normal function of the transcript needs further exploration (For comparison with other mRNA imaging methods, see Supplementary Table S1).

The formation of Pepper's functional structure requires an external factor to pull the two separated stems together. This external factor does not have to be a continuous and complementary total length of targeted mRNA. Two RNAs that are close to each other in space and interact with each other are also technically feasible. Concerning the advantages of our strategy, we speculate that this inert aptamer fluorescence system may become an available tool for the study of RNA-RNA interactions, which helps to infer the structure and target of any mRNA and long non-coding RNA. It may also help us understand the mystery of life at the level of post-transcriptional regulation.

## DATA AVAILABILITY

*NUPACK* is a growing software suite for the analysis and design of nucleic acid structures, devices, and systems (http://www.nupack.org).

*PrimerBank* is a public resource for PCR primers (https://pga.mgh.harvard.edu/primerbank).

*The Human Protein Atlas* is a Swedish-based program with the aim to map all the human proteins in cells, tissues, and organs using an integration of various omics technologies (https://www.proteinatlas.org).

## Supplementary Material

gkac368_Supplemental_Files
