# Supplementary material for: Inert Pepper aptamer-mediated endogenous mRNA recognition and imaging in living cells
Source: Nucleic Acids Res. 2022 May 17;50(14):e84. doi: 10.1093/nar/gkac368 (PMC9371900; doi:10.1093/nar/gkac368)
Supplement: gkac368_Supplemental_Files [file gkac368_supplemental_files.zip › NAR-SI (Revised 4).pdf]

Supplementary Information

Inert Pepper aptamer-mediated endogenous mRNA recognition and imaging in living cells

Qi Wang[1,†], Feng Xiao[1,†], Haomiao Su[1,4,†], Hui Liu[1], Jinglei Xu[1], Heng Tang[1], Shanshan Qin[1], Zhentian Fang[1], Ziang Lu[1], Jian Wu[3], Xiaocheng Weng[1], Xiang Zhou[1,2,*]

[1]Key Laboratory of Biomedical Polymers of Ministry of Education , College of Chemistry and Molecular Sciences, Wuhan University, Luojiashan Street, Wuchang District, Wuhan, HuBei, 430072 (P. R. China)

[2]The Institute of Advanced Studies, Wuhan University, Luojiashan Street, Wuchang District, Wuhan, HuBei, 430072 (P. R. China)

[3]School of Medicine, Wuhan University, Luojiashan Street, Wuchang District, Wuhan, HuBei, 430072 (P. R. China)

[4]Department of Chemistry, Yale University, 225 Prospect Street, New Haven, CT 06520 (USA)

[†] Joint Authors

A

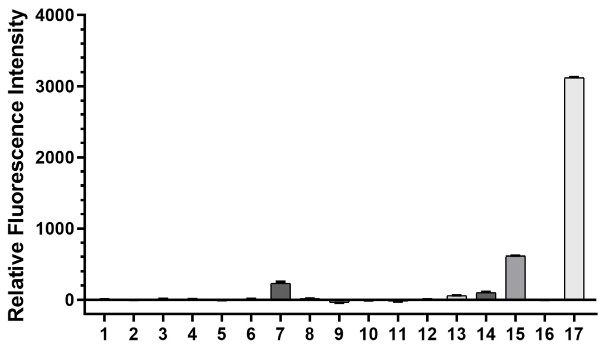

	Split Broccoli														iPepper		
	1	2	3	4	5	6	7	8	9	10	11	12	13	14	15	16	17
S1 (1 μM)	+			+		+	+	+			+		+	+			
S2 (1 μM)		+			+	+	+		+			+	+	+			
iPepper (1 μM)															+		+
Target RNA (2 μM)			+	+	+		+			+	+	+		+		+	+
DFHBI-1T (20 μM)	+	+	+	+	+	+	+										
DFHBI-1T (1 μM)								+	+	+	+	+	+	+			
HBC (1 μM)															+	+	+

B C

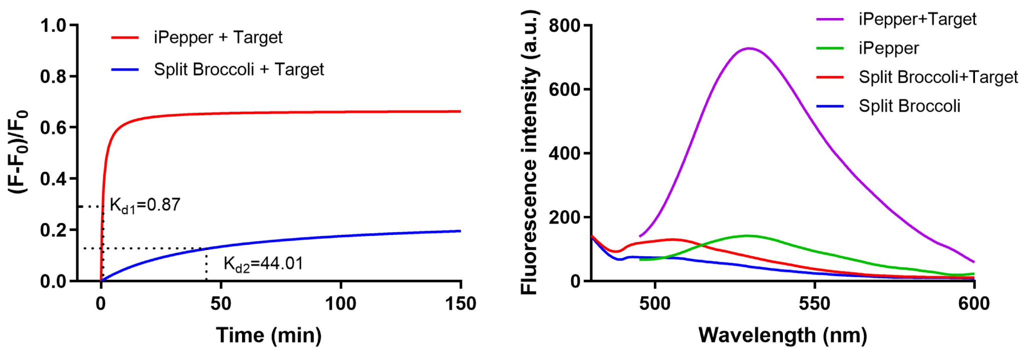

Figure S1 Comparison between Split Broccoli system and iPepper system *in vitro*. **A.** The fluorescence intensity of Split Broccoli probe and iPepper probe in imaging buffer with and without target RNA after annealing from 65°C to 25°C. Error bars are standard deviations in three repetitive assays. **B.** Fluorescence kinetics of the binding reaction between the aptamer and target RNA at 37°C. The process was equipped with the FAM scanning channel. The dissociation constant of iPepper, K_{d1} = 0.87, the dissociation constant of Split Broccoli, K_{d2} = 44.01. **C.** Fluorescence spectra of Split Broccoli probe and iPepper probe in the presence or absence of target RNA after incubation at 37°C for 18 h.

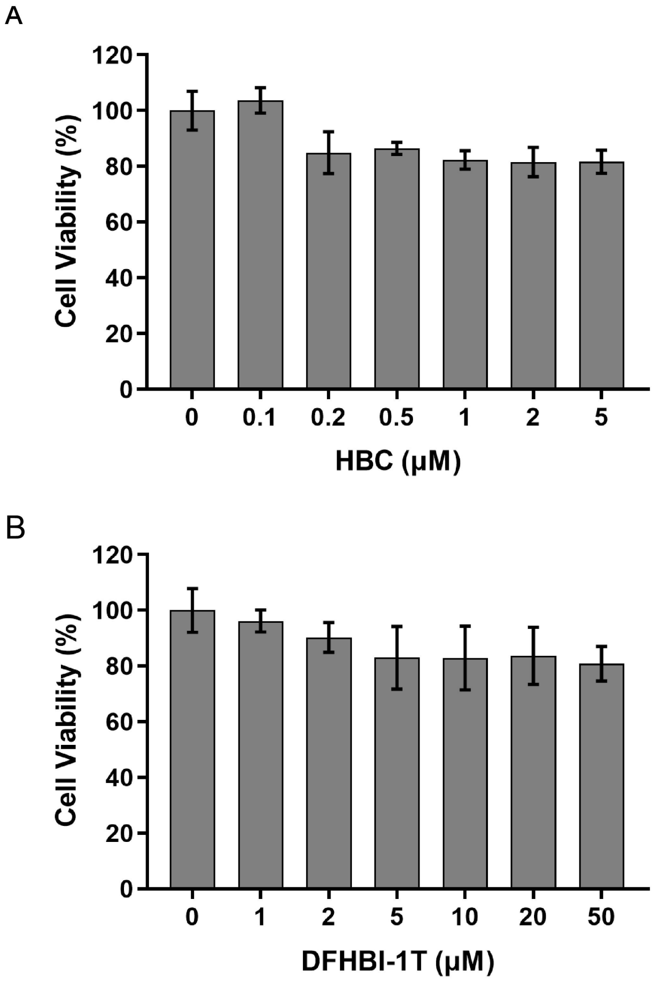

Figure S2 Cytotoxicity assays of HBC and DFHBI-1T in HeLa cells. HeLa cell viability was measured in the presence of different concentrations of the HBC (A) and DFHBI-1T (B). Data represent the mean ± s.d. from three cell cultures. HBC and DFHBI-1T showed comparable cytotoxicity at the concentration of the working solution in live cells. Error bars are standard deviations in three repetitive assays.

A

target RNA	Sequence
full-match	UCCUGAGCG CA AGUACUCCG
5' mismatch	**AGGACUCGC** CA AGUACUCCG
3' mismatch	UCCUGAGCG CA **UCAUGAGGC**
5' &3' 4nt mismatch	UCCUGAG**UU** CA **UU**UACUCCG
5' &3' 7nt mismatch	UCCUG**UUUU** CA **UUU**U**U**CUCCG
iPep III- β-actin	**CGGAGUACU**GGCACUGGCGCCGAGGGCUUCCUCCAAUCGUGGCGUGUCGGCC**CGCUCAGGA**

B

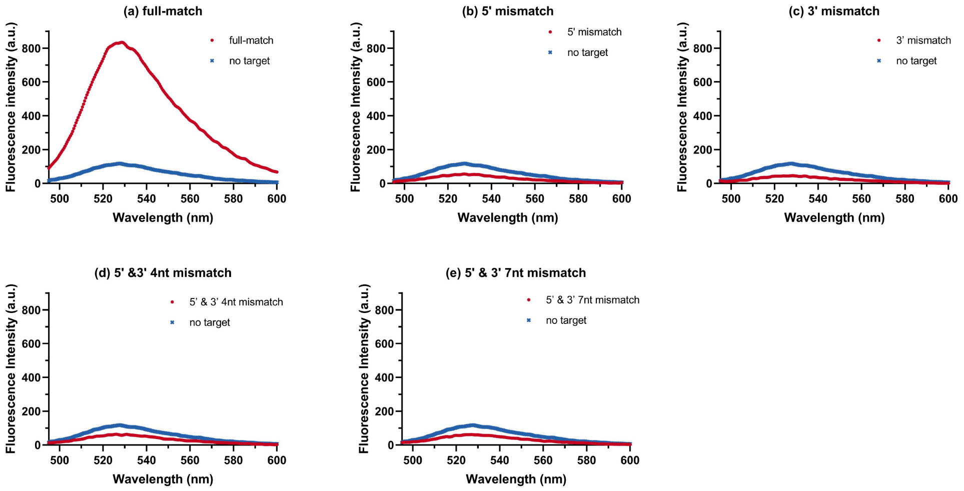

Figure S3 Selective RNA recognition by iPepper aptamer. **A.** The nucleotide sequences of target RNAs and the iPepper aptamer. The region recognized by iPepper aptamer is shown in red. Mismatched sites for the recognition region in the target RNA sequences are labeled in blue. **B.** Fluorescence spectra of iPepper in the presence (red) or absence (blue) of target RNA. The target RNA samples were (a) full-match, (b) 5'-mismatch, (c) 3'-mismatch, (d) 5'- & 3' 4 nt-mismatch, and (e) 5'- & 3' 7 nt-mismatch. For B, three independent experiments were carried out with similar results.

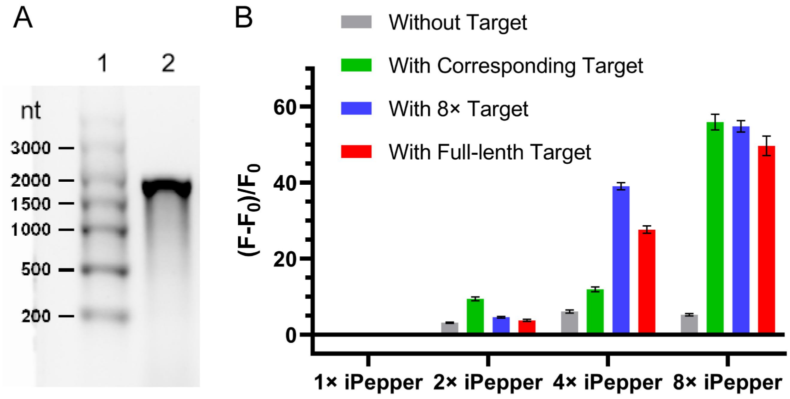

Figure S4 Combination of different tandem arrays of iPepper and target RNA with different lengths. **A.** Agarose gel electrophoresis analysis for the full-length *TUBB3* mRNA transcribed *in vitro*. Lane 1, RNA marker. Lane 2, *TUBB3* mRNA. **B.** Relative fluorescent intensity of different tandem arrays of iPepper (iPep-*TUBB3*) aptamer in the presence and absence of target RNA with different lengths. Error bars are standard deviations in three repetitive assays.

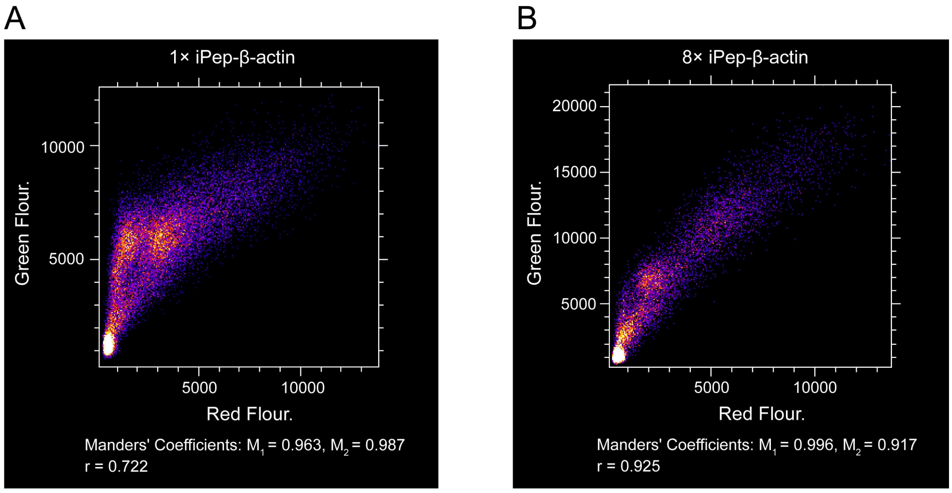

Figure S5 Colocalization analysis of Cy3-labeled probe (red fluorescence) and iPepper aptamer (green fluorescence), 1× iPep-*β-actin* (A) and 8× iPep-*β-actin* (B), in Fig. 4C. The Manders' colocalization coefficient data (1× iPep-*β-actin*: M_1 = 0.963, M_2 = 0.987, 8× iPep-*β-actin*: M_1 = 0.996, M_2 = 0.917) showed a remarkable colocalization relationship between the two fluorescent signals. M_1 and M_2 represent the proportion of the part colocalized between the iPepper aptamer and the Cy3-labeled probe to the total amount of the iPepper aptamer or the Cy3-labeled probe. r represents the Pearson Correlation Coefficient.

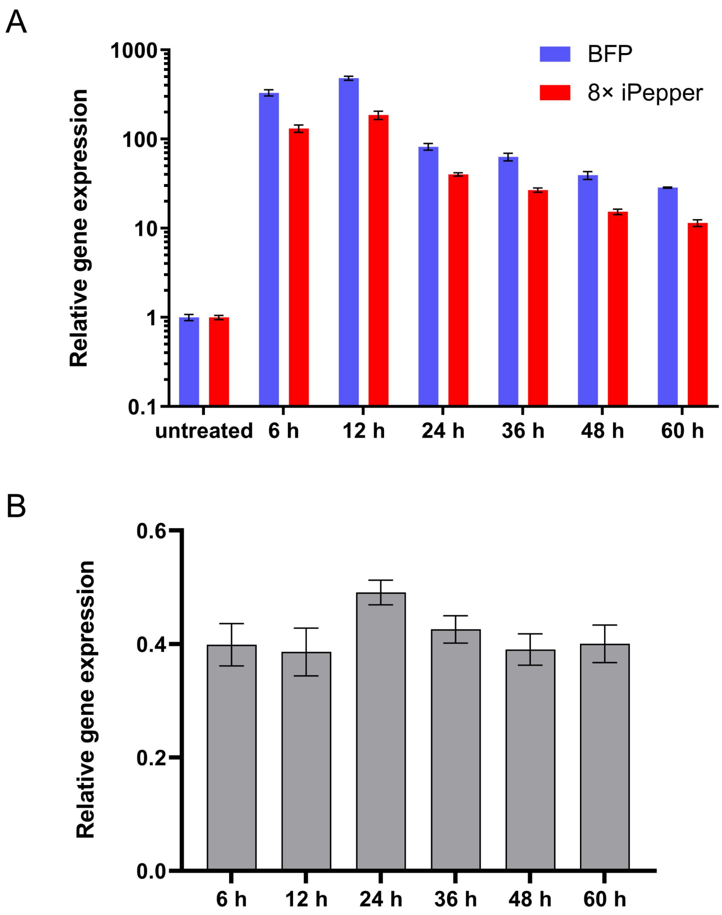

Figure S6 The relative gene expression levels of 8× iPep-*β-actin* RNA. **A.** Time-dependent gene expression levels of 8× iPep-*β-actin* and BFP relative to *GAPDH* in HeLa cells transfected with the vectors encoding BFP and 8× iPep-*β-actin*. **B.** Time-dependent gene expression levels of 8× iPep-*β-actin* relative to BFP in HeLa cells transfected with the vectors encoding BFP and 8× iPep-*β-actin*. Error bars are standard deviations in three repetitive assays.

A

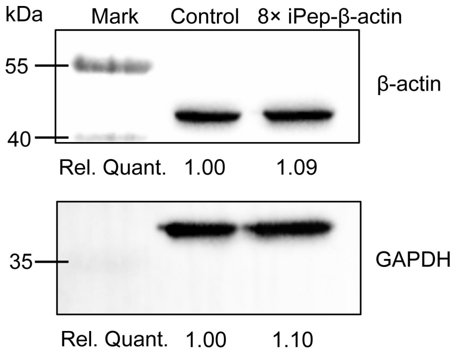

B

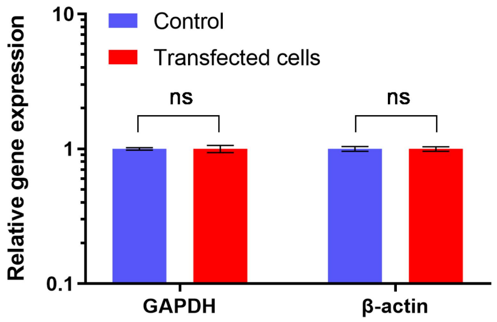

Figure S7 The effects on expression of *β-actin* upon transfection of 8× iPep-*β-actin* expressing vectors. **A.** Protein levels of *β-actin* in HEK293T cells transfected with vectors encoding 8× iPep-*β-actin* were analyzed by western blot. *GAPDH* was included as an internal reference control. **B.** The gene expression levels of *β-actin* RNA relative to *GAPDH* in HEK293T cells transfected with vectors encoding 8× iPep-*β-actin* aptamers. HEK293T cells were chosen for the experiments because of their high transfection efficiency to avoid subtle differences caused by iPepper from not being detected to a certain extent. A two-tailed t-test was performed with $\alpha = 0.05$. NS means not significant. Error bars are standard deviations in three repetitive assays.

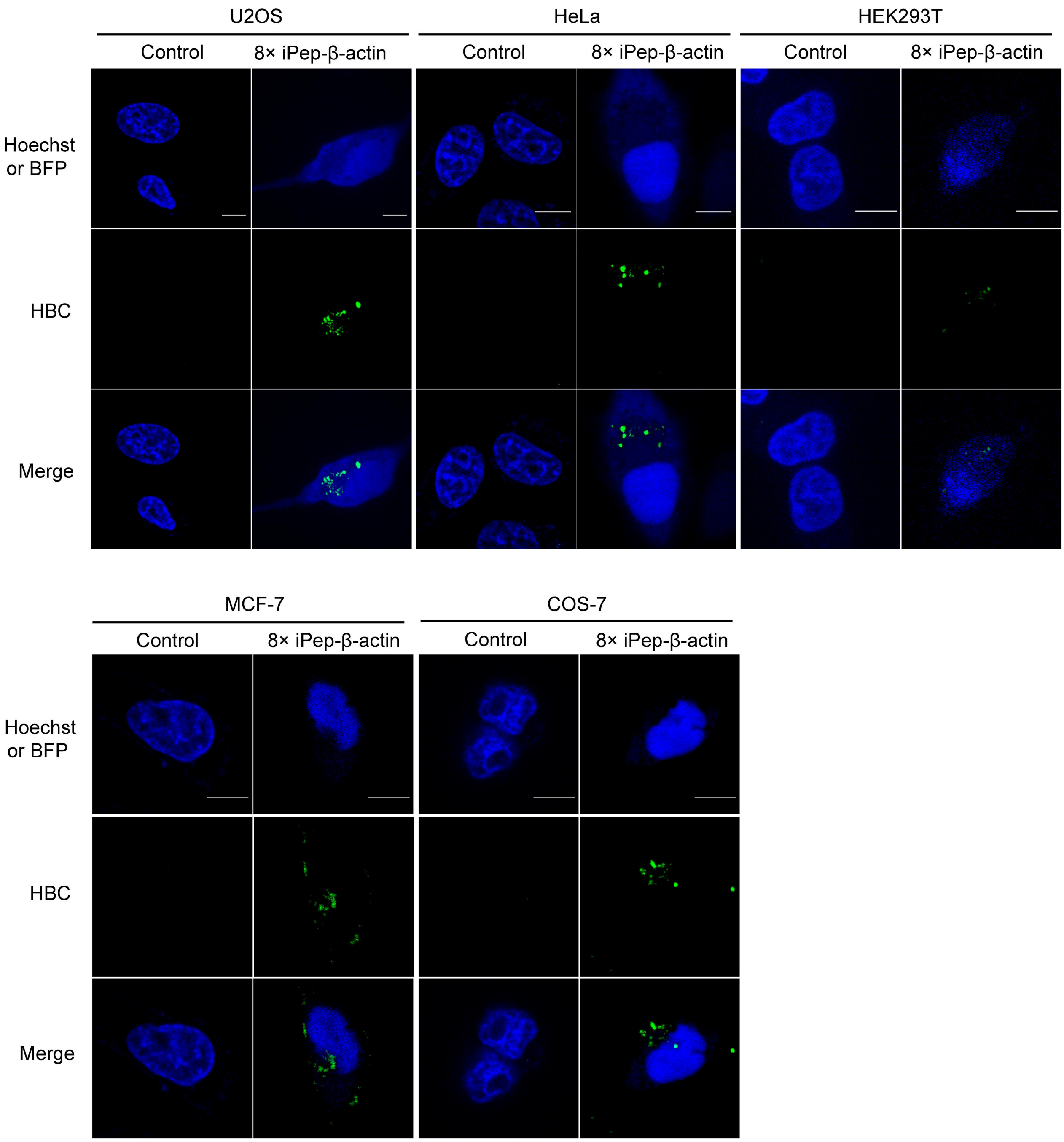

Figure S8 Live cell imaging in U2OS, HeLa, HEK293T, MCF-7, COS-7 cells transfected with vectors encoding 8× iPep-*β-actin* aptamers respectively. The nucleus is shown in blue (Hoechst 33342 or BFP). Scale bar = 10 μm. At least two independent experiments were carried out with similar results.

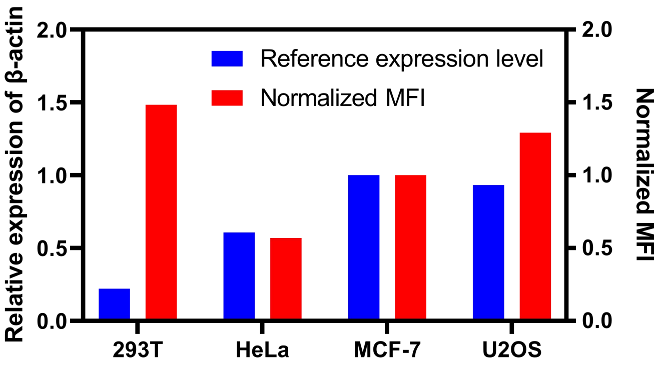

Figure S9 The mean fluorescence intensities (MFI) of cell populations in flow cytometry of different cells expressing 8× iPep-*β-actin* and the relative expression of *β-actin* mRNA in corresponding cells. Reference values are referred to The Human Protein Atlas (https://www.proteinatlas.org/). The *β-actin* expression levels and MFI are both normalized with corresponding data in MCF-7 cells.

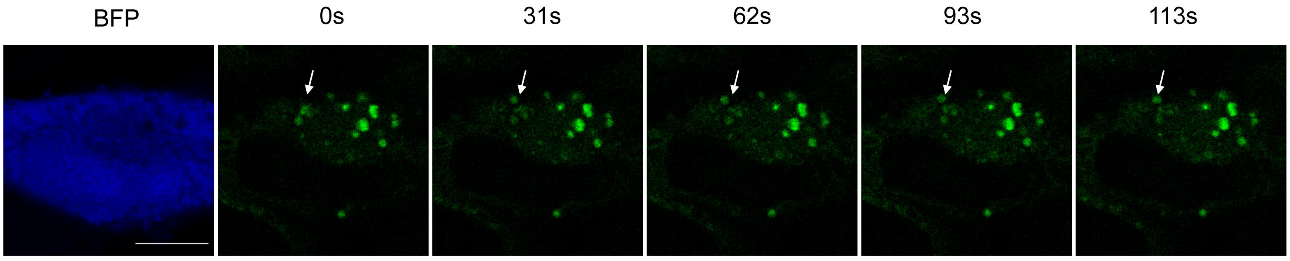

Figure S10 Real-time imaging of *β-actin* mRNA in HeLa cells using 8× iPepper (also see Video S). Time-lapse images exhibited mRNA particles (arrows) moving in restricted areas of the cytoplasm. The fluorescence of BFP marked for expression of the iPepper aptamer. Scale bar = 10 μm.

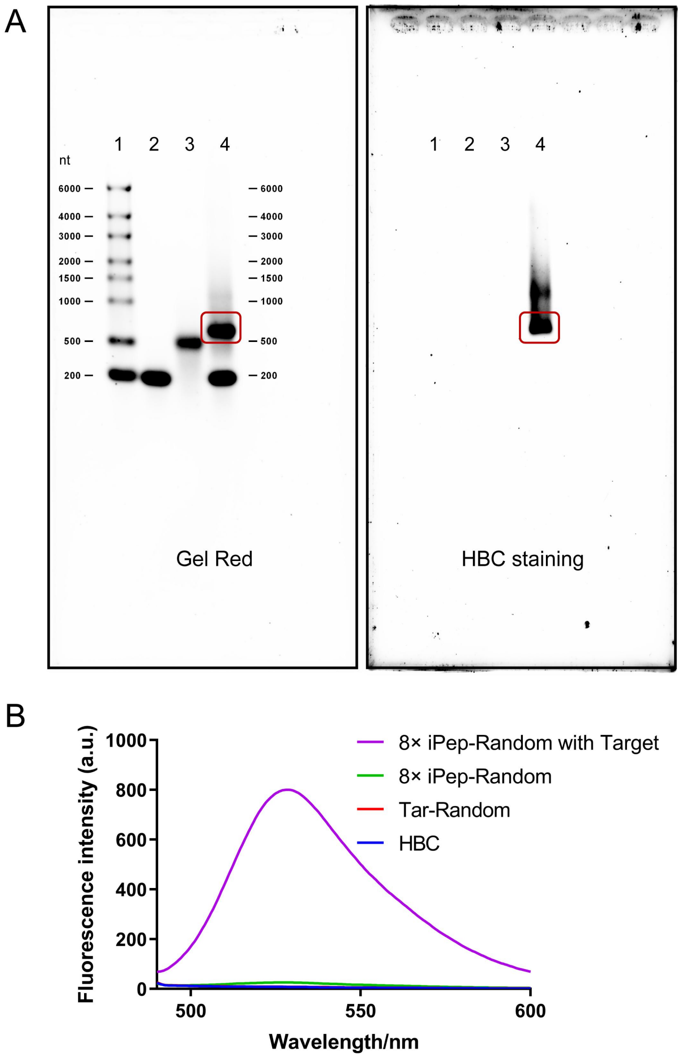

Figure S11 Characterization of 8× iPep-Random aptamers. **A.** Agarose gel electrophoresis analysis for the 8× iPep-Random binding with target RNA. Lane 1, RNA marker. Lane 2, target-Random. Lane 3, 8× iPep-Random. Lane 4, 8× iPep-Random with target-Random. Gels were stained first with HBC (right) and subsequently with Gel Red (left). **B.** Fluorescence spectra of 8× iPep-Random aptamer with and without target-Random in the presence of HBC. Three independent experiments were carried out with similar results.

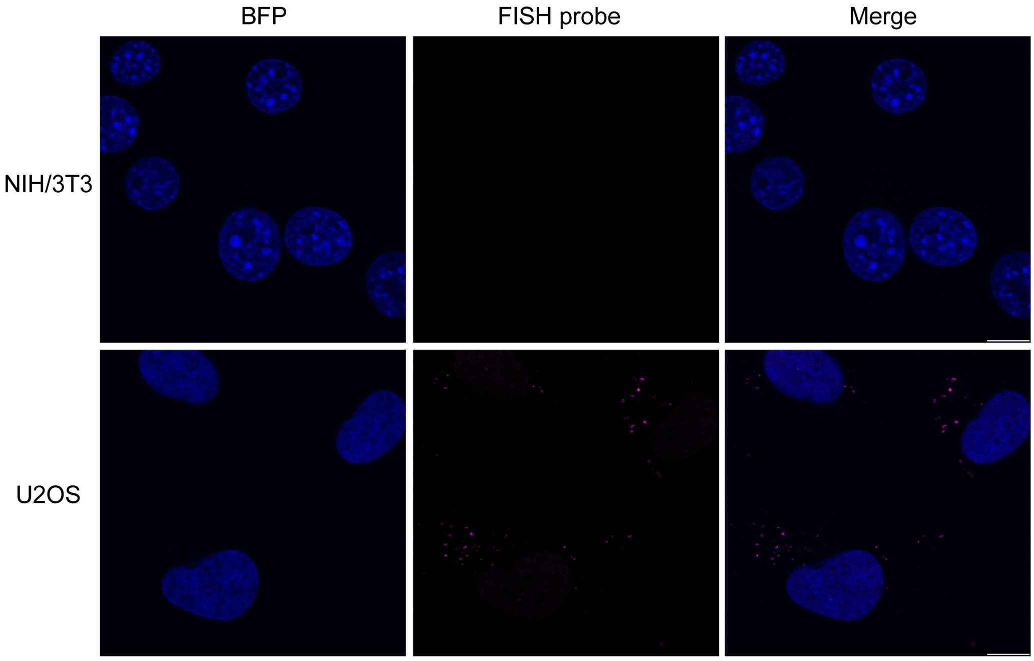

Figure S12 FISH of *NORAD* in NIH/3T3 and U2OS cells. The nucleus is shown in blue (DAPI). Scale bar = 10 µm. Two independent experiments were carried out with similar results.

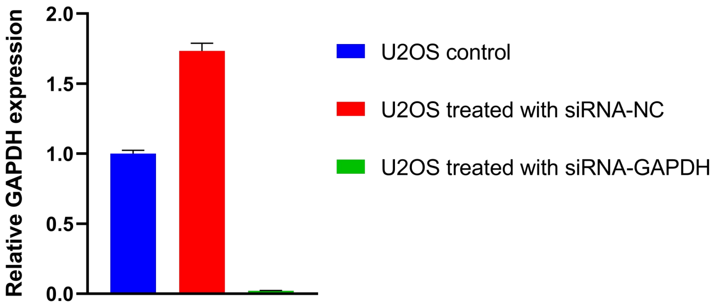

Figure S13 The relative expression levels of *GAPDH* mRNA in normal U2OS cells (blue), U2OS cells transfected with siRNA targeting to *GAPDH* mRNA(green) and U2OS cells transfected with negative control siRNA (red). The gene expression levels are normalized with *β-actin* in normal U2OS cells. Error bars are standard deviations in three repetitive assays.

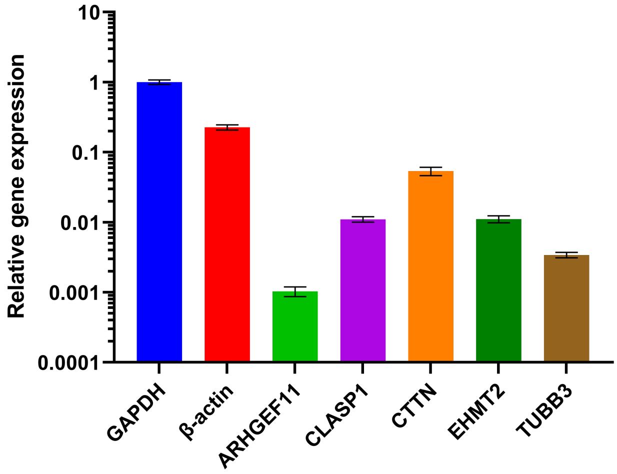

Figure S14 The relative expression levels of the seven genes that were focused in this study. The gene expression levels of *β-actin*, *ARHGEF11*, *CLASP1*, *CTTN*, *EHMT2*, and *TUBB3* are normalized with *GAPDH* in U2OS cells. Error bars are standard deviations in three repetitive assays.

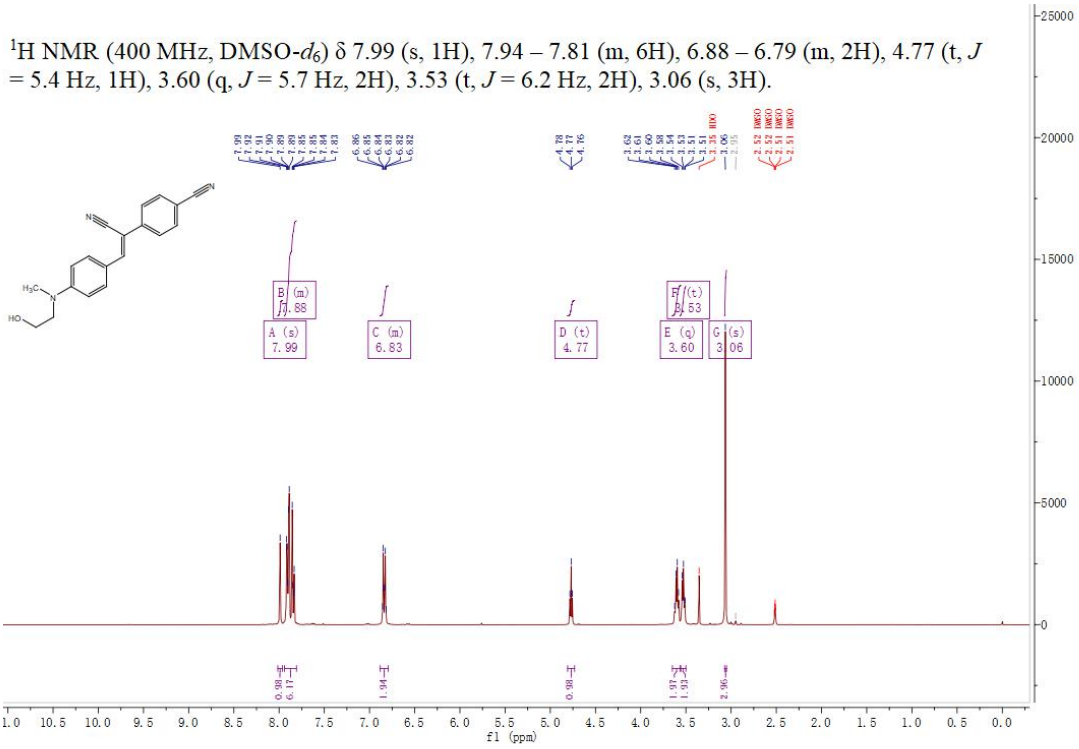

Figure S15 ^{1}H NMR (400 MHz, DMSO) spectrum of HBC.

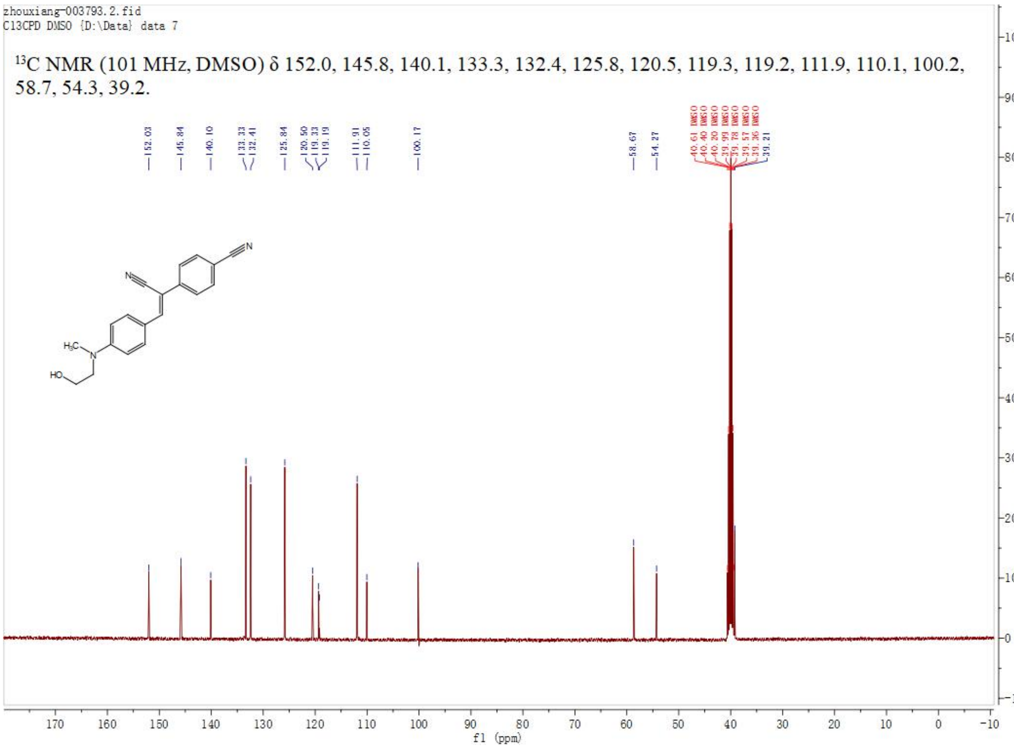

Figure S16 ^{13}C NMR (101 MHz, DMSO) spectrum of HBC.

Table S1 Comparison of different methods to image mRNA in living cells

	Component	endogenous mRNA Imaging	Delivery method	Specificity	Signal to noise	Strength	Drawbacks	References
**Directly labeled mRNAs**	Fluorescently labeled mRNA strand	No	Microinjection or transfection	Excellent	Excellent	The observed fluorescence signal only comes from the mRNA of interest, good temporal resolution	Exogenously injected mRNA interferes severely with cells, easily cause severe cell damage, unable to image endogenous mRNA	1, 2
**Molecular beacons**	Short hairpin DNA strand with a fluorophore and a quencher	Yes	Microinjection, transfection or peptide-linked	Excellent	Good	Simple structure, easy to prepare, good specificity	Difficult delivery, need to modify the chain, easy to leak and cause background fluorescence	3-5
**Nano Molecular beacons**	Molecular beacons with nanoparticles	Yes	Transfection, special receptor-mediated endocytosis	Excellent	Excellent	Low background signals due to great quenching efficiency, excellent resistance to enzyme degradation, high cellular uptake without additional transfecting agents due to special receptor-mediated endocytosis	Require dual fluorophore labeling and more complex probe design, because each probe must contain a self-complementary region and a target region	6-11
**RNA binding protein-fluorescent protein system**	A fusion protein of a fluorescent protein and a specific sequence binding protein, and an MS2 binding sequence needs to be conjugated to the mRNA of interest	No	Transfection	Good	Good	Genetically encoded, no fluorophore modification required, excellent system to track mRNAs from transcription to translation	The mRNP complex is large and needs to modify the mRNA of interest, free fusion protein is easy to produce background fluorescence	12-20
**CRISPR/Cas9-fluorescent protein system**	CRISPR/Cas9 and fluorescent protein system	Yes	Transfection	Excellent	Good	Good targeting specificity, no need to modify the mRNA of interest	Strict restrictions on the mRNA sequence, need to carefully design the sgRNA sequence to avoid unexpected DNA gene editing, resulting in cytotoxicity	21-23
**RNA aptamer/fluorophore system**	RNA aptamers and the corresponding fluorophores	No	Transfection	Excellent	Good	Genetically encoded, small size, suitable for real-time tracking of RNAs	Need to modify the mRNA of interest	24-32
**Our method**	Inert Pepper aptamers (iPeppers) and HBC dye	Yes	Transfection	Excellent	Excellent	Genetically encoded, small size, suitable for real-time tracking of RNAs, high signal-to-noise ratio, high target accessibility	Need to carefully design the probe sequence to produce the highest signal-to-noise ratio	

Table S2 Sequence list of RNA aptamer and target

Name	Sequence (5'-3')
Pepper I	AUCGCCCAAUCGUGGCGUGUCGGCCUUUCGAGGCACUGGCGCCGGCGAU
Pepper II	GAUCGCACUGGCGCCGCCAAUCGUGGCGUGUCGGCGAUC
Pepper III	GAUCGCACUGGCGCCGGAGGGCUUCCUCCCAAUCGUGGCGUGUCGGCGAUC
Pepper III-6nt	**GAUCGC**ACUGGCGCCGGAGGGCUUCCUCCCAAUCGUGGCGUGUCG**GCGAUC**
Pepper III-4nt	**GAUC**ACUGGCGCCGGAGGGCUUCCUCCCAAUCGUGGCGUGUCG**GAUC**
Pepper III-3nt	**GGC**ACUGGCGCCGGAGGGCUUCCUCCCAAUCGUGGCGUGUCG**GCC**
Pepper III-2nt	**GC**ACUGGCGCCGGAGGGCUUCCUCCCAAUCGUGGCGUGUCG**GC**
iPep III-*β-actin* (iPep-*β-actin*)	CGGAGUACUGGCACUGGCGCCGAGGGCUUCCUCCAAUCGUGGCGUGUCGGCCCGCUCAGGA
iPep I-*β-actin*	CGGAGUACUGGCCCAAUCGUGGCGUGUCGGCCUUUCGAGGCACUGGCGCCAGCCCGCUCAGGA
Target RNA (for 1× iPep-*β-actin*)	UCCUGAGCGCAAGUACUCCG
Target DNA (for 1× iPep-*β-actin*)	TCCTGAGCGCAAGTACTCCG
Split Broccoli 1 (S1)	GGAGACGGUCGGGUCCAGUAUGCUCAGGAGG
Split Broccoli 2 (S2)	CACGGAGUACGUACUGUCGAGUAGAGUGUGGGCUCC
Target RNA (for Split Broccoli)	CCUCCUGAGCGCAAGUACUCCGUG
1× iPepper / Target	CGGAGUACUGGCACUGGCGCCGAGGGCUUCCUCCAAUCGUGGCGUGUCGGCCCGCUCAGGA / UCCUGAGCGCAAGUACUCCG
2× iPepper / Target	GCGCUCAGGAGGCACUGGCGCCGAGGGCUUCCUCCAAUCGUGGCGUGUCGGCCGGAGCAAUGAUCUUGAUCUAGGCACUGGCGCCGAGGGCUUCCUCCAAUCGUGGCGUGUCGGCCGAUUGUGCUG / CAGCACAAUGAAGAUCAAGAUCAUUGCUCCUCCUGAGCGC

4× iPepper / Target	GCGAGGCCAAGGCACUGGCGCCGAGGGCUUCCUCCAAUCGUGGCGUGUCGGCCGAUGGAGC<br>CGCCGAUCCACAGGCACUGGCGCCGAGGGCUUCCUCCAAUCGUGGCGUGUCGGCCGGGAGU<br>ACUUGCGCUCAGGAGGCACUGGCGCCGAGGGCUUCCUCCAAUCGUGGCGUGUCGGCCGGAG<br>CAAUGAUCUUGAUCUAGGCACUGGCGCCGAGGGCUUCCUCCAAUCGUGGCGUGUCGGCCGA<br>UUGUGCUG /<br>CAGCACAAUGAAGAUCAAGAUCAUUGCUCCUCCUGAGCGCAAGUACUCCGUGUGGAUCGGC<br>GGCUCCAUCCUGGCCUCGC
Target RNA<br>(for 8× iPep-*β-actin*)	CAGCACAAUGAAGAUCAAGAUCAUUGCUCCUCCUGAGCGCAAGUACUCCGUGUGGAUCGGC<br>GGCUCCAUCCUGGCCUCGCUGUCCACCUUCCAGCAGAUGUGGAUCAGCAAGCAGGAGUAUG<br>ACGAGUCCGGCCCCUCCAUCGUCCACCGCAAAUGCUUC
1× iPepper-*TUBB3* /<br>Target	AAGCAGGGUAGGCACUGGCGCCGAGGGCUUCCUCCAAUCGUGGCGUGUCGGCCGCGGCAGC<br>AA / UUGCUGCCGACACCCUGCUU
2× iPepper-*TUBB3* /<br>Target	AACGUCAGGAGGCACUGGCGCCGAGGGCUUCCUCCAAUCGUGGCGUGUCGGCCGUGGAGCU<br>GCAAUAAGACAAGGCACUGGCGCCGAGGGCUUCCUCCAAUCGUGGCGUGUCGGCCGGACAG<br>GAGC / GCUCCUGUCUCUGUCUUAUUGCAGCUCCAGGCCUGACGUU
4× iPepper-*TUBB3* /<br>Target	AACGUCAGGAGGCACUGGCGCCGAGGGCUUCCUCCAAUCGUGGCGUGUCGGCCGUGGAGCU<br>GCAAUAAGACAAGGCACUGGCGCCGAGGGCUUCCUCCAAUCGUGGCGUGUCGGCCGGACAG<br>GAGCAGCUCACACAGGCACUGGCGCCGAGGGCUUCCUCCAAUCGUGGCGUGUCGGCCGGGC<br>CUAGGUGGGGAGGACAGGCACUGGCGCCGAGGGCUUCCUCCAAUCGUGGCGUGUCGGCCGG<br>GCCAUAAA /<br>UUUAUGGCCUCGUCCUCCCCACCUAGGCCACGUGUGAGCUGCUCCUGUCUCUGUCUUAUUG<br>CAGCUCCAGGCCUGACGUU
8× Target RNA<br>(for 8× iPep-*TUBB3*)	UCUAAACCCCCGGAGCCAUCUUGCUGCCGACACCCUGCUUUCCCCUCGCCCUAGGGCUCCCU<br>UGCCGCCCUCCUGCAGUAUUUAUGGCCUCGUCCUCCCCACCUAGGCCACGUGUGAGCUGCUC<br>CUGUCUCUGUCUUAUUGCAGCUCCAGGCCUGACGUU
Target RNA-Random	GGAAUGGUAGACAUGCGGUGGCUCACACUAUUCCCUCCACAUCAACCCCAAUACUUAAACC<br>GCCUCCAAAUUCCAAUAUACUAAAUCAUCACCACUCCCAACACACAUACACAACACUAACCC<br>AAACUCACUCAAUUCUCUUCCCCUUUCACAUCCAUUU

Table S3 Sequence list of DNA nucleotide

Application	Name	Sequence (5'-3')
FISH	*β-actin*-Cy3 probe	Cy3-CGTCGCCCACATAGGAATCCTTCTG
		Cy3-CTGCTGTCACCTTCACCGTTCCAGT
	TUBB3-Cy5 probe	Cy5-ATAGGAACATTCTGGCCTAGAACCC
		Cy5-TTCCATCTAGAAGGGCTAGATGTGA
qPCR	*β-actin*-FP	CTCGCCTTTGCCGATCC
	β-actin-RP	TCTCCATGTCGTCCCAGTTG
	GAPDH-FP	AATGGGCAGCCGTTAGGAAA
	GAPDH-RP	GCGCCCAATACGACCAAATC
	CTTN-FP	GTGGTTTTGGCGGCAAGTATG
	CTTN-RP	CTCTCTGTGACTCGTGCTTCT
	TUBB3-FP	GGCCAAGGGTCACTACACG
	TUBB3-FP	GCAGTCGCAGTTTTCACACTC
	EHMT2-FP	GGGCGGGAAAATCACCTCC
	EHMT2-RP	CTGTCAGAGGAGTTAGGTTCTGC
	CLASP1-FP	CTTGCTGTCGGAGTCCACG
	CLASP1-RP	CTCTCTGTGACTCGTGCTTCT
	ARHGEF11-FP	ATGAGTGTAAGGTTACCCCAGAG
	ARHGEF11-FP	CGTTGAACGAGACCTGTTGT
	BFP-FP	AGAAAACACTCGGCTGGGAG
	BFP-RP	TGCTAGGGAGGTCGCAGTAT
	8× iPep-*β-actin*-FP	GGACTCGTCATACTCAGGCAC
	8× iPep-*β-actin*-RP	CCAGTGCCTCTCGCTGTC
Transcription	T7 primer	CGGTAATACGACTCACTATAGG
	Pepper I	CGGTAATACGACTCACTATAGGGATCGCCCAATCGTGGCGTGTCGGCCT TTCGAGGCACTGGCGCCGGCGAT
	Pepper I-C	ATCGCCGGCGCCAGTGCCTCGAAAGGCCGACACGCCACGATTGGGCGA TCCCTATAGTGAGTCGTATTACCG
	Pepper II	CGGTAATACGACTCACTATAGGGATCGCACTGGCGCCGCCAATCGTGGC GTGTCGGCGATC
	Pepper II-C	GATCGCCGACACGCCACGATTGGCGGCGCCAGTGCGATCCCTATAGTG AGTCGTATTACCG

	Pepper III	CGGTAATACGACTCACTATAGGGATCGCACTGGCGCCGGAGGGCTTCCTCCCAATCGTGGCGTGTCGGCGATC
	Pepper III-C	GATCGCCGACACGCCACGATTGGGAGGAAGCCCTCCGGCGCCAGTGCGATCCCTATAGTGAGTCGTATTACCG
	Pepper III-6nt	CGGTAATACGACTCACTATAGGGATCGCACTGGCGCCGGAGGGCTTCCTCCCAATCGTGGCGTGTCGGCGATC
	Pepper III-6nt-C	GATCGCCGACACGCCACGATTGGGAGGAAGCCCTCCGGCGCCAGTGCGATCCCTATAGTGAGTCGTATTACCG
	Pepper III-4nt	CGGTAATACGACTCACTATAGGGATCACTGGCGCCGGAGGGCTTCCTCCCAATCGTGGCGTGTCGGATC
	Pepper III-4nt-C	GATCCGACACGCCACGATTGGGAGGAAGCCCTCCGGCGCCAGTGATCCCTATAGTGAGTCGTATTACCG
	Pepper III-3nt	CGGTAATACGACTCACTATAGGGATCGGCACTGGCGCCGGAGGGCTTCCTCCCAATCGTGGCGTGTCGGCC
	Pepper III-3nt-C	GGCCGACACGCCACGATTGGGAGGAAGCCCTCCGGCGCCAGTGCCGATCCCTATAGTGAGTCGTATTACCG
	Pepper III-2nt	CGGTAATACGACTCACTATAGGGATCGCACTGGCGCCGGAGGGCTTCCTCCCAATCGTGGCGTGTCGGC
	Pepper III-2nt-C	GCCGACACGCCACGATTGGGAGGAAGCCCTCCGGCGCCAGTGCGATCCCTATAGTGAGTCGTATTACCG
	iPep III-*β-actin*	CGGTAATACGACTCACTATAGGGATCGGAGTACTGGCACTGGCGCCGAGGGCTTCCTCCAATCGTGGCGTGTCGGCCCGCTCAGGA
	iPep III-*β-actin*-C	TCCTGAGCGGGCCGACACGCCACGATTGGAGGAAGCCCTCGGCGCCAGTGCCAGTACTCCGATCCCTATAGTGAGTCGTATTACCG
	iPep I-*β-actin*	CGGTAATACGACTCACTATAGGGATCGGAGTACTGGCCCAATCGTGGCGTGTCGGCCTTTCGAGGCACTGGCGCCAGCCCGCTCAGGA
	iPep I-*β-actin*-C	TCCTGAGCGGGCTGGCGCCAGTGCCTCGAAAGGCCGACACGCCACGATTGGGCCAGTACTCCGATCCCTATAGTGAGTCGTATTACCG
	Split Broccoli 1	CGGTAATACGACTCACTATAGGAGACGGTCGGGTCCAGTATGCTCAGGAGG

	Split Broccoli 1-C	CCTCCTGAGCATACTGGACCCGACCGTCTCCTATAGTGAGTCGTATTACCG
	Split Broccoli 2	CGGTAATACGACTCACTATAGGCACGGAGTACGTACTGTCGAGTAGAGTGTGGGCTCC
	Split Broccoli 2-C	GGAGCCCACACTCTACTCGACAGTACGTACTCCGTGCCTATAGTGAGTCGTATTACCG

Note: Sequence of 8× iPepper RNA aptamers

F30-8× Pepper

UUGCCAUGUGUAUGUGGGUUCGCCCACAUACUCUGAUGAUCCCCAAUCGUGGCGUGUCGGCCUCUCCCAAUCGUGGCG
UGUCGGCCUCUCCCAAUCGUGGCGUGUCGGCCUCUCCCAAUCGUGGCGUGUCGGCCUCUCCCAAUCGUGGCGUGUCGG
CCUCUCCCAAUCGUGGCGUGUCGGCCUCUCCCAAUCGUGGCGUGUCGGCCUCUCCCAAUCGUGGCGUGUCGGCCUCUCU
UCGGAGAGGCACUGGCGCCGGAGAGGCACUGGCGCCGGAGAGGCACUGGCGCCGGAGAGGCACUGGCGCCGGAGAGGC
ACUGGCGCCGGAGAGGCACUGGCGCCGGAGAGGCACUGGCGCCGGAGAGGCACUGGCGCCGGGAUCAUUCAUGGCAA

8× iPep-β-actin

GAAGCAUUUUGGCACUGGCGCCGAGGGCUUCCUCCAAUCGUGGCGUGUCGGCCUGGUGGACGAUGGAGGGGCAGGCAC
UGGCGCCGAGGGCUUCCUCCAAUCGUGGCGUGUCGGCCAGACUCGUCAUACUCCUGCAGGCACUGGCGCCGAGGGCUU
CCUCCAAUCGUGGCGUGUCGGCCAGCUGAUCCACAUCUGCUGUGGCACUGGCGCCGAGGGCUUCCUCCAAUCGUGGCG
UGUCGGCCAAGGUGGACAGCGAGGCCAAGGCACUGGCGCCGAGGGCUUCCUCCAAUCGUGGCGUGUCGGCCAAUGGAG
CCGCCGAUCCACAGGCACUGGCGCCGAGGGCUUCCUCCAAUCGUGGCGUGUCGGCCAGGAGUACUUGCGCUCAGGAGG
CACUGGCGCCGAGGGCUUCCUCCAAUCGUGGCGUGUCGGCCAGAGCAAUGAUCUUGAUCUUGGCACUGGCGCCGAGGG
CUUCCUCCAAUCGUGGCGUGUCGGCCUAUUGUGCUG

8× iPep-*CTTN*

GAUCAAAAAAGGCACUGGCGCCGAGGGCUUCCUCCAAUCGUGGCGUGUCGGCCGGUUUAUUCUGAAUUCUUUAGGCAC
UGGCGCCGAGGGCUUCCUCCAAUCGUGGCGUGUCGGCCGUUUAAAAAAAUCAUACCUAGGCACUGGCGCCGAGGGCUU
CCUCCAAUCGUGGCGUGUCGGCCGGAGGUGUGCUACAGGAAUAGGCACUGGCGCCGAGGGCUUCCUCCAAUCGUGGCG
UGUCGGCCGAGAUACAAUAAGUUGCAUAGGCACUGGCGCCGAGGGCUUCCUCCAAUCGUGGCGUGUCGGCCGAAAACC
CGACCUCAUUGCAGGCACUGGCGCCGAGGGCUUCCUCCAAUCGUGGCGUGUCGGCCGAUUGUGGUAAAGCAAGGAAGG
CACUGGCGCCGAGGGCUUCCUCCAAUCGUGGCGUGUCGGCCGAUGAGAAAAUGCACCUCAAGGCACUGGCGCCGAGGG
CUUCCUCCAAUCGUGGCGUGUCGGCCGAGCAAAAAC

8× iPep-GAPDH

GUACAUGACAGGCACUGGCGCCGAGGGCUUCCUCCAAUCGUGGCGUGUCGGCCGGGUGCGGCUCCCUAGGCCAGGCAC
UGGCGCCGAGGGCUUCCUCCAAUCGUGGCGUGUCGGCCGUCCCCUCUUCAAGGGGUCAGGCACUGGCGCCGAGGGCUU
CCUCCAAUCGUGGCGUGUCGGCCGCAUGGCAACUGUGAGGAGAGGCACUGGCGCCGAGGGCUUCCUCCAAUCGUGGCG
UGUCGGCCGGAGAUUCAGUGUGGUGGGAGGCACUGGCGCCGAGGGCUUCCUCCAAUCGUGGCGUGUCGGCCGACUGAG
UGUGGCAGGGACAGGCACUGGCGCCGAGGGCUUCCUCCAAUCGUGGCGUGUCGGCCGCCCAGCAGUGAGGGUCUCAGG
CACUGGCGCCGAGGGCUUCCUCCAAUCGUGGCGUGUCGGCCGUCUUCCUCUUGUGCUCUUAGGCACUGGCGCCGAGGG
CUUCCUCCAAUCGUGGCGUGUCGGCCGUGGGGCUGG

8× iPep-*TUBB3*

AACGUCAGGAGGCACUGGCGCCGAGGGCUUCCUCCAAUCGUGGCGUGUCGGCCGUGGAGCUGCAAUAAGACAAGGCAC
UGGCGCCGAGGGCUUCCUCCAAUCGUGGCGUGUCGGCCGGACAGGAGCAGCUCACACAGGCACUGGCGCCGAGGGCUU
CCUCCAAUCGUGGCGUGUCGGCCGGGCCUAGGUGGGGAGGACAGGCACUGGCGCCGAGGGCUUCCUCCAAUCGUGGCG
UGUCGGCCGGGCCAUAAAUACUGCAGGAGGCACUGGCGCCGAGGGCUUCCUCCAAUCGUGGCGUGUCGGCCGGGCGGC
AAGGGAGCCCUAAGGCACUGGCGCCGAGGGCUUCCUCCAAUCGUGGCGUGUCGGCCGGCGAGGGGAAAGCAGGGUAGG
CACUGGCGCCGAGGGCUUCCUCCAAUCGUGGCGUGUCGGCCGCGGCAGCAAGAUGGCUCCAGGCACUGGCGCCGAGGG
CUUCCUCCAAUCGUGGCGUGUCGGCCGGGGUUUAGA

8× iPep-*EHMT2*

CUGACUGAUAGGCACUGGCGCCGAGGGCUUCCUCCAAUCGUGGCGUGUCGGCCACCUGACUCCUCAUCUUCCAGGCAC
UGGCGCCGAGGGCUUCCUCCAAUCGUGGCGUGUCGGCCAUUCUUCUUCCUCUUCCUCAGGCACUGGCGCCGAGGGCUU
CCUCCAAUCGUGGCGUGUCGGCCACCUCUUCCUCUUCUUCUUAGGCACUGGCGCCGAGGGCUUCCUCCAAUCGUGGCG
UGUCGGCCAUCCUCCUCUUCCUCCUCCAGGCACUGGCGCCGAGGGCUUCCUCCAAUCGUGGCGUGUCGGCCACUCUUCA
CUUAGUUGUUCAGGCACUGGCGCCGAGGGCUUCCUCCAAUCGUGGCGUGUCGGCCAUUAGAGCUUCAACUUCAGAGGC
ACUGGCGCCGAGGGCUUCCUCCAAUCGUGGCGUGUCGGCCAUUGCUGUCGGAGUCCACGAGGCACUGGCGCCGAGGGC
UUCCUCCAAUCGUGGCGUGUCGGCCACUCAUCCAC

8× iPep-*CLASP1*

CAAAGUUCUAGGCACUGGCGCCGAGGGCUUCCUCCAAUCGUGGCGUGUCGGCCAUUUGCUGUCCUUUAAAGUAGGCAC
UGGCGCCGAGGGCUUCCUCCAAUCGUGGCGUGUCGGCCAAACAUUACGCUUGAUAAAAGGCACUGGCGCCGAGGGCUU
CCUCCAAUCGUGGCGUGUCGGCCAAAGGGUGGCAGGGAAAGAAGGCACUGGCGCCGAGGGCUUCCUCCAAUCGUGGCG
UGUCGGCCAAAGACUGAUGAGACAAAGAGGCACUGGCGCCGAGGGCUUCCUCCAAUCGUGGCGUGUCGGCCAAGUUGC
AUUACCUGCACAAGGCACUGGCGCCGAGGGCUUCCUCCAAUCGUGGCGUGUCGGCCAGUGGUAAGCUAGUGAGGCAGG
CACUGGCGCCGAGGGCUUCCUCCAAUCGUGGCGUGUCGGCCAAUAGAAAAGGCAGGCCCGAGGCACUGGCGCCGAGGG
CUUCCUCCAAUCGUGGCGUGUCGGCCAGCAUCGCCG

8× iPep-*ARHGEF11*

ACUUAACCUAGGCACUGGCGCCGAGGGCUUCCUCCAAUCGUGGCGUGUCGGCCACUAUACUCUGGGGUAACCAGGCAC
UGGCGCCGAGGGCUUCCUCCAAUCGUGGCGUGUCGGCCAACACUCAUGGUUUCUCGGAGGCACUGGCGCCGAGGGCUU
CCUCCAAUCGUGGCGUGUCGGCCAUCUCCACGGUUCCAGAAUAGGCACUGGCGCCGAGGGCUUCCUCCAAUCGUGGCG
UGUCGGCCAUGUAGCUUUGGUGUCUUGAGGCACUGGCGCCGAGGGCUUCCUCCAAUCGUGGCGUGUCGGCCACAGGAU
UGAAUCGCUUGCAGGCACUGGCGCCGAGGGCUUCCUCCAAUCGUGGCGUGUCGGCCAUUUUUGAGCAAGGUGAGAAGG
CACUGGCGCCGAGGGCUUCCUCCAAUCGUGGCGUGUCGGCCAAGGGCCUCCCAACUUGAAAGGCACUGGCGCCGAGGG
CUUCCUCCAAUCGUGGCGUGUCGGCCAUAGAAUCCU

8× iPep-*NORAD*

CACAGGCCUAGGCACUGGCGCCGAGGGCUUCCUCCAAUCGUGGCGUGUCGGCCACAUAAACGGCCAGUAAAUAGGCAC
UGGCGCCGAGGGCUUCCUCCAAUCGUGGCGUGUCGGCCAUCCCAGAGGGUGGUGGGCCGGCACUGGCGCCGAGGGCUU
CCUCCAAUCGUGGCGUGUCGGCCAUUCCAACGGGCCAAACGUAGGCACUGGCGCCGAGGGCUUCCUCCAAUCGUGGCG
UGUCGGCCACCUGUCAUUCUACCAUUUAGGCACUGGCGCCGAGGGCUUCCUCCAAUCGUGGCGUGUCGGCCACUCUUC
CGACAGCAAAGUAGGCACUGGCGCCGAGGGCUUCCUCCAAUCGUGGCGUGUCGGCCAGGUAGAAUGAAGACCAACAGG
CACUGGCGCCGAGGGCUUCCUCCAAUCGUGGCGUGUCGGCCACCCGAUGGCCGCUAACCGAGGCACUGGCGCCGAGGG
CUUCCUCCAAUCGUGGCGUGUCGGCCACCACCCGUC

8× iPep-Random

AAAUGGAUGAGGCACUGGCGCCGAGGGCUUCCUCCAAUCGUGGCGUGUCGGCCAAAAGGGGAAGAGAAUUGAAGGCA
CUGGCGCCGAGGGCUUCCUCCAAUCGUGGCGUGUCGGCCAGAGUUUGGGUUAGUGUUGAGGCACUGGCGCCGAGGGCU
UCCUCCAAUCGUGGCGUGUCGGCCAUAUGUGUGUUGGGAGUGGAGGCACUGGCGCCGAGGGCUUCCUCCAAUCGUGGC
GUGUCGGCCAAUGAUUUAGUAUAUUGGAAGGCACUGGCGCCGAGGGCUUCCUCCAAUCGUGGCGUGUCGGCCAUUGGA
GGCGGUUUAAGUAAGGCACUGGCGCCGAGGGCUUCCUCCAAUCGUGGCGUGUCGGCCAGGGGUUGAUGUGGAGGGAA
GGCACUGGCGCCGAGGGCUUCCUCCAAUCGUGGCGUGUCGGCCAAGUGUGAGCCACCGCAUGAACCACUGGCGCCGAG
GGCUUCCUCCAAUCGUGGCGUGUCGGGUAUACCAUUCC

Reference

1. Cha B., Koppetsch B.S., Theurkauf W.E. In Vivo Analysis of Drosophila bicoid mRNA Localization Reveals a Novel Microtubule-Dependent Axis Specification Pathway. *Cell* 2001; 106:35-46.

2. Tadakuma H., Ishihama Y., Shibuya T., Tani T., Funatsu T. Imaging of single mRNA molecules moving within a living cell nucleus. *Biochem. Bioph. Res. Co.* 2006; 344:772-779.

3. Tyagi S., Kramer F.R. Molecular Beacons: Probes that Fluoresce upon Hybridization. *Nat. Biotechnol.* 1996; 14:303-308.

4. Tsourkas A., Behlke M.A., Bao G. Structure-function relationships of shared-stem and conventional molecular beacons. *Nucleic Acids Res.* 2002; 30:4208-4215.

5. Wang K., Tang Z., Yang C.J., Kim Y., Fang X., Li W., Wu Y., Medley C.D., Cao Z., Li J. et al. . Molecular Engineering of DNA: Molecular Beacons. *Angew Chem. Int. Ed.* 2009; 48:856-870.

6. Yang Y., Huang J., Yang X., Quan K., Wang H., Ying L., Xie N., Ou M., Wang K. FRET Nanoflares for Intracellular mRNA Detection: Avoiding False Positive Signals and Minimizing Effects of System Fluctuations. *J. Am. Chem. Soc.* 2015; 137:8340-8343.

7. Yang Y., Huang J., Yang X., Quan K., Wang H., Ying L., Xie N., Ou M., Wang K. FRET Nanoflares for Intracellular mRNA Detection: Avoiding False Positive Signals and Minimizing Effects of System Fluctuations. *J. Am. Chem. Soc.* 2015; 137:8340-8343.

8. Briley W.E., Bondy M.H., Randeria P.S., Dupper T.J., Mirkin C.A. Quantification and real-time tracking of RNA in live cells using Sticky-flares. *Proc. Natl. Acad. Sci. U.S.A.* 2015; 112:9591.

9. Riahi R., Wang S., Long M., Li N., Chiou P., Zhang D.D., Wong P.K. Mapping Photothermally Induced Gene Expression in Living Cells and Tissues by Nanorod-Locked Nucleic Acid Complexes. *ACS Nano* 2014; 8:3597-3605.

10. Wang S., Riahi R., Li N., Zhang D.D., Wong P.K. Single Cell Nanobiosensors for Dynamic Gene Expression Profiling in Native Tissue Microenvironments. *Adv. Mater.* 2015; 27:6034-6038.

11. Seferos D.S., Giljohann D.A., Hill H.D., Prigodich A.E., Mirkin C.A. Nano-Flares: Probes for Transfection and mRNA Detection in Living Cells. *J. Am. Chem. Soc.* 2007; 129:15477-15479.

12. Bertrand E., Chartrand P., Schaefer M., Shenoy S.M., Singer R.H., Long R.M. Localization of ASH1 mRNA Particles in Living Yeast. *Mol. Cell* 1998; 2:437-445.

13. Park H.Y., Lim H., Yoon Y.J., Follenzi A., Nwokafor C., Lopez-Jones M., Meng X., Singer R.H. Visualization of Dynamics of Single Endogenous mRNA Labeled in Live Mouse. *Science* 2014; 343:422-424.

14. Carrocci T.J., Hoskins A.A. Imaging of RNAs in live cells with spectrally diverse small molecule fluorophores. *Analyst 2014*; 139:44-47.

15. Han S., Zhao B.S., Myers S.A., Carr S.A., He C., Ting A.Y. RNA-protein interaction mapping via MS2- or Cas13-based APEX targeting. *Proc. Natl. Acad. Sci. U.S.A.* 2020; 117:22068-22079.

16. Hu C., Chinenov Y., Kerppola T.K. Visualization of Interactions among bZIP and Rel Family Proteins in Living Cells Using Bimolecular Fluorescence Complementation. *Mol. Cell* 2002; 9:789-798.

17. Hu C.D., Kerppola T.K. Simultaneous visualization of multiple protein interactions in living cells using multicolor fluorescence complementation analysis. *Nat. Biotechnol.* 2003; 21:539-545.

18. Chen H., Yan Z., Shang Y., Lin H., Zhou J.M. Firefly Luciferase Complementation Imaging Assay for Protein-Protein Interactions in

Plants. *Plant Physiol.* 2008; 146:368-376.

19. Walter M., Chaban C., Schutze K., Batistic O., Kudla J. Visualization of protein interactions in living plant cells using bimolecular fluorescence complementation. *Plant J.* 2010; 40:428-438.

20. Wu B., Chen J., Singer R.H. Background free imaging of single mRNAs in live cells using split fluorescent proteins. *Sci. Rep.-UK* 2015; 4.

21. Yang L., Wang Y., Li S., Yao R., Luan P., Wu H., Carmichael G.G., Chen L. Dynamic Imaging of RNA in Living Cells by CRISPR-Cas13 Systems. *Mol. Cell* 2019; 76:981-997.

22. Chen B., Gilbert L.A., Cimini B.A., Schnitzbauer J., Zhang W., Li G., Park J., Blackburn E.H., Weissman J.S., Qi L.S. et al. . Dynamic Imaging of Genomic Loci in Living Human Cells by an Optimized CRISPR/Cas System. *Cell* 2013; 155:1479-1491.

23. Nelles D.A., Fang M.Y., O Connell M.R., Xu J.L., Markmiller S.J., Doudna J.A., Yeo G.W. Programmable RNA Tracking in Live Cells with CRISPR/Cas9. *Cell* 2016; 165:488-496.

24. Paige J.S., Wu K.Y., Jaffrey S.R. RNA Mimics of Green Fluorescent Protein. *Science* 2011; 333:642-646.

25. Han K.Y., Leslie B.J., Fei J., Zhang J., Ha T. Understanding the Photophysics of the Spinach-DFHBI RNA Aptamer-Fluorogen Complex To Improve Live-Cell RNA Imaging. *J. Am. Chem. Soc.* 2013; 135:19033-19038.

26. Filonov G.S., Moon J.D., Svensen N., Jaffrey S.R. Broccoli: Rapid Selection of an RNA Mimic of Green Fluorescent Protein by Fluorescence-Based Selection and Directed Evolution. *J. Am. Chem. Soc.* 2014; 136:16299-16308.

27. Song W., Strack R.L., Svensen N., Jaffrey S.R. Plug-and-Play Fluorophores Extend the Spectral Properties of Spinach. *J. Am. Chem. Soc.* 2014; 136:1198-1201.

28. Song W., Filonov G.S., Kim H., Hirsch M., Li X., Moon J.D., Jaffrey S.R. Imaging RNA polymerase III transcription using a photostable RNA-fluorophore complex. *Nat. Chem. Biol.* 2017; 13:1187-1194.

29. Wirth R., Gao P., Nienhaus G.U., Sunbul M., Jäschke A. SiRA: A Silicon Rhodamine-Binding Aptamer for Live-Cell Super-Resolution RNA Imaging. *J. Am. Chem. Soc.* 2019; 141:7562-7571.

30. Chen X., Zhang D., Su N., Bao B., Xie X., Zuo F., Yang L., Wang H., Jiang L., Lin Q. et al. Visualizing RNA dynamics in live cells with bright and stable fluorescent RNAs. *Nat. Biotechnol.* 2019; 37:1287-1293.

31. Li X., Kim H., Litke J.L., Wu J., Jaffrey S.R. Fluorophore-Promoted RNA Folding and Photostability Enables Imaging of Single Broccoli-Tagged mRNAs in Live Mammalian Cells. *Angew Chem. Int. Ed.* 2020; 59:4511-4518.

32. Li X., Mo L., Litke J.L., Dey S.K., Suter S.R., Jaffrey S.R. Imaging Intracellular S-Adenosyl Methionine Dynamics in Live Mammalian Cells with a Genetically Encoded Red Fluorescent RNA-Based Sensor. *J. Am. Chem. Soc.* 2020; 142:14117-14124.
