# Supplementary material for: Inert Pepper aptamer-mediated endogenous mRNA recognition and imaging in living cells
Source: Nucleic Acids Res. 2022 May 17;50(14):e84. doi: 10.1093/nar/gkac368 (PMC9371900; doi:10.1093/nar/gkac368)
Supplement: gkac368_Supplemental_Files [file gkac368_supplemental_files.zip › Supplementary video legends.docx]

Supplementary video legends:

Dynamic imaging of β-actin mRNA in HeLa cells by the iPepper system.

Imaging results show the phenomenon of mRNA particles moving in restricted regions of the cytoplasm.
